# Epigenetic Memory and Hormonal Crosstalk in Plant Drought Adaptation: Mechanisms, miRNAs, and Technological Advances

**DOI:** 10.3390/epigenomes10030052

**Published:** 2026-08-06

**Authors:** Emanuela Talarico, Eleonora Greco, Marina Camoli, Francesco Guarasci, Cristina Teruzzi, Fabrizio Araniti, Leonardo Bruno

**Affiliations:** 1 Unit of Plant Biology, Department of Biology, Ecology and Earth Sciences (DiBEST), University of Calabria, 87036 Arcavacata of Rende, Italy; emanuela.talarico@unical.it (E.T.); eleonora.greco@unical.it (E.G.); marina.camoli@unical.it (M.C.); francesco.guarasci@unical.it (F.G.); 2 Department of Agricultural and Environmental Sciences—Production, Landscape, Agroenergy (Di.S.A.A.), University of Milano, 20133 Milano, Italy; cristina.teruzzi@unimi.it (C.T.); fabrizio.araniti@unimi.it (F.A.)

**Keywords:** drought stress, epigenetic memory, DNA methylation, histone modifications, MicroRNAs (miRNAs), hormonal signalling, transgenerational inheritance, crop resilience

## Abstract

Drought poses a major threat to global food security, making it critical to understand the molecular mechanisms underlying plant responses to water scarcity. Epigenetic modifications, including DNA methylation and histone alterations, play central roles in regulating genes and hormonal pathways essential for drought adaptation. MicroRNAs, while primarily functioning as post-transcriptional regulators, can also influence epigenetic pathways and contribute to chromatin remodelling, suggesting a role in modulating epigenetic memory. Investigating these interactions is essential for understanding how plants integrate epigenetic and post-transcriptional regulation during stress. Epigenetic memory in drought-adapted plants provides insights into the transgenerational inheritance of adaptive traits and reveals how plants balance genome stability with flexibility. The crosstalk between epigenetic mechanisms and hormonal signalling is crucial for fine-tuning gene expression, promoting drought resilience. This review proposes a conceptual framework integrating epigenetic, hormonal, and miRNA-mediated regulation of drought responses. It emphasizes the impact of advanced technologies, such as bisulfite sequencing and CRISPR-Cas9, in dissecting plant epigenetic responses to drought. These approaches improve our understanding of drought tolerance mechanisms and offer promising strategies for developing resilient crops for sustainable agriculture. However, direct evidence linking epitranscriptomic modifications to long-term drought memory remains limited, and this emerging regulatory layer requires further experimental validation.

## 1. Introduction

Increasing frequency and severity of drought events associated with ongoing climate change represent a major constraint on agricultural productivity worldwide. Water deficit affects plant growth, development, and reproduction, ultimately reducing crop yields and threatening global food security. As drought episodes become more frequent and prolonged, understanding the molecular mechanisms that enable plants to withstand water limitation has become a central objective for the development of resilient cropping systems [1,2,3].

Plant adaptation to drought relies on the coordinated action of multiple regulatory processes operating across physiological, transcriptional, and molecular levels. Although considerable advances have been made in characterising individual drought-response pathways, the integration of epigenetic and hormonal regulation into a unified framework remains incompletely understood. This gap is particularly relevant given the central role of these mechanisms in determining stress perception, signal transduction, and adaptive responses to water deficit [4,5].

Among the molecular processes involved in drought adaptation, epigenetic regulation has emerged as a major determinant of stress-responsive gene expression, with DNA methylation being recognized as one of the most extensively studied mechanisms involved in the regulation of plant responses to environmental stresses [6]. DNA methylation and histone modifications can dynamically alter chromatin architecture, thereby influencing transcriptional activity and cellular responses to environmental stimuli [7,8,9]. At the same time, phytohormones act as primary signalling molecules that translate environmental cues into physiological and developmental responses [10,11]. Rather than acting independently, hormonal and epigenetic pathways increasingly appear to operate as interconnected regulatory modules that jointly shape drought responses.

An additional level of complexity is provided by the phenomenon of epigenetic memory, whereby previous stress exposure influences subsequent plant responses. Evidence accumulated over the last decade indicates that drought-induced changes in DNA methylation and histone modifications may persist beyond the initial stress event, contributing to sustained transcriptional states and enhanced stress resilience [4,12,13,14,15]. The mechanisms underlying the establishment, maintenance, and biological significance of this memory remain active areas of investigation.

In this review, we focus on the interactions among epigenetic modifications, hormonal signalling pathways, and small RNA-mediated regulation in plant drought responses. Particular attention is devoted to the role of epigenetic memory and to the molecular mechanisms through which these regulatory layers contribute to stress adaptation across diverse plant species.

We also discuss how recent technological advances are transforming the study of drought-associated epigenetic processes. The development of high-throughput sequencing approaches, chromatin profiling methods, and targeted genome and epigenome editing technologies has significantly expanded our capacity to investigate the regulatory networks underlying plant responses to water deficit.

Although several recent reviews have examined individual aspects of plant stress epigenetics, including DNA methylation, histone modifications, epigenetic memory, or small RNA-mediated regulation, the present review provides a unified conceptual framework integrating these regulatory layers with hormonal crosstalk and emerging technological advances in the specific context of drought adaptation. Particular emphasis is placed on the mechanistic interactions among these pathways and on their potential application in crop improvement. This review primarily covers advances reported during the last decade while also including seminal studies that established the foundations of the field.

To integrate the diverse regulatory layers described above, we propose a conceptual framework in which drought response emerges from the dynamic interplay between epigenetic modifications, hormonal signalling, and small RNA-mediated regulation [16,17,18,19,20]. Under water deficit conditions, stress perception triggers rapid hormonal responses—primarily mediated by abscisic acid (ABA)—which initiate downstream transcriptional reprogramming [18,19,20,21]. Concurrently, epigenetic mechanisms such as DNA methylation and histone modifications modulate chromatin accessibility, thereby stabilising or fine-tuning the expression of stress-responsive genes [16,17,19,21,22,23].

Within this framework, microRNAs (miRNA) act as critical intermediaries, linking post-transcriptional regulation with both hormonal signalling and epigenetic control [16,19,22,24,25]. By targeting transcription factors, hormone biosynthesis genes, and epigenetic regulators, miRNAs contribute to shaping both immediate stress responses and longer-term adaptive processes [16,24,25,26,27,28].

Importantly, these regulatory layers are interconnected through feedback loops. Hormonal signals (including ABA) can influence the establishment of epigenetic marks, while epigenetic states can modulate hormone sensitivity and signalling efficiency [16,19,20,26,28]. This bidirectional crosstalk enables plants to balance rapid responsiveness with the establishment of stress memory [17,18,26,27,29].

We further distinguish between two functional phases of drought adaptation: (i) a short-term response characterised by reversible transcriptional and hormonal changes, and (ii) a long-term adaptive phase involving epigenetic memory, which may persist after stress recovery and, in some cases, across generations [18,26,27,29]. Notably, the extent to which drought-induced “memory” is meiotically stable and consistently transgenerational—and the mechanisms by which such memory might evade resetting—remain incompletely resolved and appear species-/context-dependent [18,26,29]. This integrative model provides a unifying perspective for understanding how plants coordinate molecular responses to drought stress and highlights key regulatory nodes for potential biotechnological intervention (e.g., epigenome editing and ncRNA-centered strategies) [16,25,26].

In the following sections, this framework serves as a guiding structure for interpreting how individual regulatory layers, DNA methylation, histone modifications, and small RNA pathways contribute to both short-term stress responses and long-term adaptive memory in plants exposed to drought.

Because *Arabidopsis* and rice currently provide the largest body of mechanistic evidence regarding drought-associated epigenetic regulation, these species are discussed more extensively throughout the review. Nevertheless, examples from additional crop species are included whenever available to broaden the translational relevance of the concepts presented.

## 2. Epigenetic Memory, Persistence, and Transmission Under Drought Stress

Drought represents a major constraint for plant productivity, driving the evolution of complex adaptive strategies. In addition to immediate physiological and molecular responses, increasing attention has been devoted to the concept of epigenetic memory, which enables plants to retain information about previous stress exposure and adjust future responses accordingly [12,13]. Epigenetic regulation—primarily mediated by DNA methylation, histone modifications, and non-coding RNAs—plays a crucial role in modulating gene expression without altering the underlying DNA sequence, thereby contributing to plant plasticity under fluctuating environmental conditions [4,14,15].

This section examines the mechanisms underlying drought-induced epigenetic memory, with particular emphasis on its potential transgenerational transmission and on the dynamic processes that regulate the maintenance and resetting of epigenetic marks.

In this review, epigenetic memory is operationally defined as the persistence of stress-induced epigenetic states, including changes in DNA methylation, histone modifications, chromatin organization, and associated regulatory pathways, that influence subsequent responses after the initial stress has ceased, regardless of whether these states are maintained within the same individual or transmitted to subsequent generations. This definition is consistent with current concepts of plant stress memory, while recognizing that transgenerational inheritance has been demonstrated only in specific species and experimental contexts [4,12,14].

### 2.1. Establishment, Maintenance, and Resetting of Drought-Induced Epigenetic Memory

The formation of epigenetic memory requires the establishment of molecular signatures that persist after an initial stress event and influence future responses. During drought priming, plants undergo extensive epigenetic reprogramming, resulting in altered transcriptional states that facilitate a faster or more effective response upon subsequent stress exposure. Persistent DNA methylation patterns and stable histone modifications are among the principal mechanisms associated with this memory effect [4,30]. The establishment and maintenance of these epigenetic states depend on specialised enzymatic machinery. DNA methyltransferases play a central role, with DRM (Domains Rearranged Methyltransferase) mediating de novo methylation and CMT3 (Chromomethylase 3) contributing to the maintenance of methylation patterns during DNA replication [31]. Histone-modifying enzymes, including histone acetyltransferases (HATs), histone methyltransferases, and histone deacetylases (HDACs), further regulate chromatin accessibility and help sustain drought-associated transcriptional programmes [32].

Recent evidence indicates that drought memory operates through multiple regulatory layers. In maize subjected to prolonged mild drought, Forestan et al. [33] identified three categories of memory-associated genes: “transcriptional memory” genes, which maintain altered expression after recovery; “epigenetic memory candidates”, which retain stress-induced chromatin changes without persistent transcriptional alterations; and “delayed memory” genes, which store stress information and respond at later developmental stages. These observations suggest that distinct forms of memory may coexist within the same plant response. Although epigenetic marks can persist for extended periods, they are not permanently fixed. The ability to remove or remodel stress-associated modifications is essential for maintaining developmental flexibility and preventing maladaptive responses when environmental conditions change. Consequently, epigenetic resetting may occur during stress recovery or specific developmental transitions, restoring transcriptional states while preserving overall genomic responsiveness [13]. Throughout this review, resetting refers to context-dependent remodelling of stress-associated epigenetic states. Depending on the biological context, resetting may involve complete erasure, partial remodelling, or selective removal of specific epigenetic marks rather than a universal return to the pre-stress state.

This resetting process is mediated by several classes of chromatin-associated enzymes. Histone demethylases (HDMs), phosphatases (PPs), deubiquitinases (DUBs), and histone deacetylases (HDACs) contribute to the dynamic regulation of chromatin structure and gene expression by removing or modifying previously established epigenetic marks [34,35,36,37,38]. An additional component of chromatin reorganisation involves the Swi2/Snf2-Related 1 (SWR1) chromatin-remodelling complex in *Arabidopsis thaliana*. By replacing canonical histones with variant forms, SWR1 alters nucleosome composition and influences transcriptional activity. Mutations affecting SWR1 components, including *SERRATED LEAVES AND EARLY FLOWERING (SWC6)*, *SUPPRESSOR OF FRIGIDA (SUF3)* [39], and *PHOTOPERIOD-INDEPENDENT EARLY FLOWERING 1 (PIE1)*, lead to developmental abnormalities and altered stress responses, highlighting the importance of chromatin remodelling in plant adaptation [40].

Within the conceptual framework proposed in this review, drought-induced epigenetic memory represents a long-term regulatory layer that contributes to sustained adaptation following initial stress exposure.

### 2.2. Persistence and Transmission of Drought-Induced Epigenetic Modifications

A major question in plant epigenetics concerns whether stress-induced epigenetic states can persist beyond the exposed individual and influence subsequent generations.

Robust evidence for stable transgenerational inheritance remains relatively limited and is largely restricted to a small number of experimental systems, whereas many reported observations derive from single studies that require further validation across species and environmental conditions.

In this context, transgenerational epigenetic inheritance refers to the transmission of stress-associated phenotypic traits without alterations in DNA sequence. Such inheritance could enable the progeny of drought-exposed plants to display enhanced tolerance to water deficit [41,42].

Nevertheless, current evidence indicates that the transmission of drought-induced epigenetic changes is often limited and context-dependent. Studies in *Arabidopsis thaliana* have shown that drought-associated DNA methylation changes can persist within the exposed generation but are not necessarily stably inherited by descendants [43]. These findings suggest that epigenetic memory may frequently function as a transient adaptive mechanism, balancing environmental responsiveness with regulatory stability.

Histone modifications have also been implicated in the persistence of drought-associated memory. Both H3K4me3, commonly linked to transcriptional activation, and H3K27me3, generally associated with repression, have been connected to drought-responsive gene regulation. Interestingly, their effects appear to differ from those observed in developmental contexts, as repressive marks may coexist with active transcription in stress-responsive loci, potentially owing to the action of drought-induced transcription factors that counteract silencing signals [44].

At the molecular level, the maintenance and potential transmission of epigenetic states involve extensive interactions between classical epigenetic mechanisms and non-coding RNAs. Small interfering RNAs (siRNAs) and microRNAs (miRNAs) can direct DNA methylation and chromatin remodelling at specific genomic regions, resulting in transcriptional gene silencing [45]. These molecules may also be transferred through reproductive tissues, thereby contributing to the propagation of epigenetic information across generations [46,47,48]. Given the central role of reproductive structures in the transmission of biological information across generations, a deeper understanding of ovule development and tissue maturation may also contribute to future studies investigating inheritance-related processes in plants [49]. Although this example derives from developmental biology rather than drought stress, it illustrates fundamental epigenetic regulatory mechanisms that are likely to operate across multiple developmental and environmental contexts, including plant responses to abiotic stress. Long non-coding RNAs (lncRNAs) provide an additional regulatory layer by recruiting chromatin-modifying complexes to specific genomic loci. Through this mechanism, they influence chromatin organisation and gene expression patterns and may contribute to the establishment of stable epigenetic states [50,51,52]. Collectively, these observations indicate that the persistence of drought-induced epigenetic states depends on a complex interaction among DNA methylation, histone modifications, and non-coding RNAs (Table 1). However, the extent to which such modifications can be maintained across generations remains incompletely resolved and appears to vary according to species, genomic context, and environmental conditions. More broadly, environmentally induced epigenetic variation has been associated with plant responses to diverse abiotic and developmental cues, including heavy metal exposure, salinity, light-dependent growth plasticity, hormone-responsive developmental regulation, and temperature-dependent developmental responses, highlighting the broad adaptive significance of epigenetic mechanisms beyond drought stress [53,54,55,56,57].

## 3. Interplay Between Epigenetic Regulation and Hormonal Signalling Under Drought Stress: A Framework-Based Perspective

Building on the conceptual framework proposed above, this section examines how epigenetic mechanisms regulate hormone signalling pathways, with particular emphasis on their roles in coordinating rapid (short-term) responses and stabilising long-term adaptive memory under drought stress.

Drought stress triggers extensive reprogramming of plant physiological and molecular processes, largely mediated by hormone signalling networks. Increasing evidence indicates that these pathways are tightly regulated by epigenetic mechanisms, including DNA methylation, histone modifications, and small RNA-mediated processes [58,59,60,61]. Through these regulatory layers, plants can dynamically modulate the expression of hormone-related genes, enabling rapid and flexible responses to water deficit.

**Figure 1:**
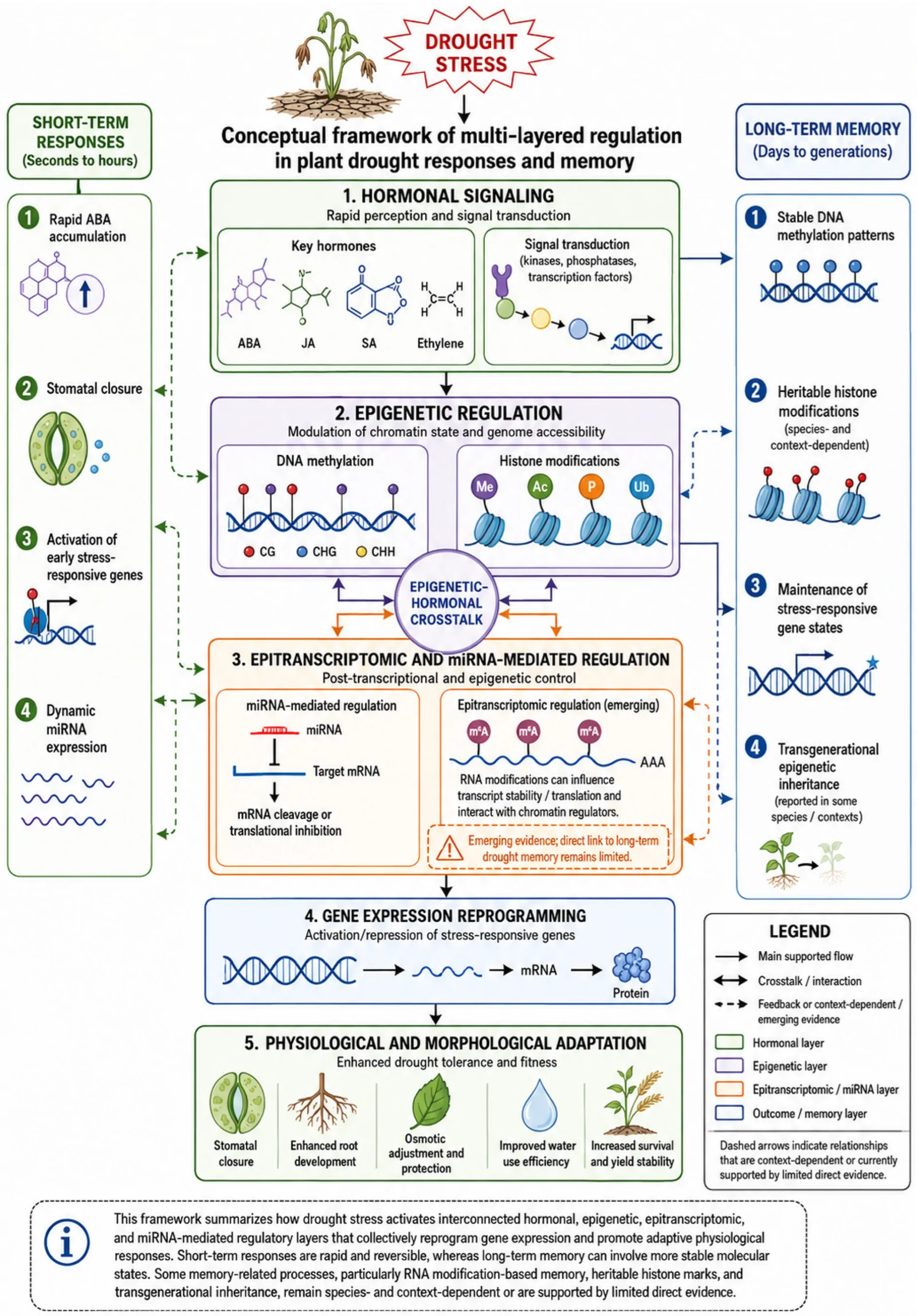
Conceptual framework of multi-layered regulation in plant drought responses integrating temporal dynamics. Drought stress activates coordinated regulatory processes operating across multiple molecular layers and timescales. Short-term responses (seconds to hours) include rapid and reversible adjustments such as stomatal closure and early gene activation, whereas long-term responses (hours or days to generations) involve more stable molecular states associated with drought memory, although some processes remain species- and context-dependent. Five interconnected regulatory layers are highlighted: (1) hormonal signalling (ABA, ethylene, jasmonates, salicylic acid), (2) chromatin-based epigenetic regulation (DNA methylation and histone modifications), (3) miRNA-mediated regulation, (4) epitranscriptomic regulation (e.g., m^6^A RNA modifications), and (5) gene expression reprogramming, which integrates these regulatory inputs before driving adaptive physiological and developmental responses. These layers interact through dynamic crosstalk and feedback loops to regulate gene expression. Their integration drives physiological and developmental responses, including stomatal regulation, root system remodelling, osmotic adjustment, and improved water use efficiency, ultimately enhancing drought tolerance and yield stability. Solid arrows indicate relationships supported by current evidence, double-headed arrows represent regulatory crosstalk, whereas dashed arrows indicate emerging, context-dependent, or currently insufficiently validated relationships. Epitranscriptomic regulation is presented as an emerging regulatory layer because direct evidence linking RNA modifications to long-term drought memory remains limited. Likewise, heritable histone modifications and transgenerational epigenetic inheritance are represented as species- and context-dependent processes rather than universally established outcomes.

A conceptual model (Figure 1) illustrates the integration between drought-induced epigenetic modifications and ABA signalling, highlighting their coordinated role in controlling stress-responsive gene expression.

### 3.1. DNA Methylation as a Regulator of Hormonal Responses to Drought

#### 3.1.1. DNA Methylation and ABA Signalling

DNA methylation is among the most extensively investigated epigenetic mechanisms involved in plant adaptation to drought stress. By regulating gene expression, methylation dynamics influence multiple aspects of phytohormone biosynthesis, signalling, and downstream responses, thereby contributing to the coordination of stress-adaptive processes [62,63]. A substantial body of evidence links DNA methylation with ABA-mediated drought responses. Analyses of the ABA-deficient maize mutant *vp10* (*viviparous 10*), characterised by premature seed germination, revealed altered responses to water deficit compared with wild-type plants, suggesting that DNA methylation contributes to the regulation of ABA-dependent stress adaptation [64]. Similarly, drought-induced methylation changes in citrus have been associated with enhanced resilience through modulation of ABA signalling pathways [65]. Additional evidence comes from maize, where drought-responsive genes such as *DRE-Binding Factor 1* (*DBF1*) are activated through ABA-dependent pathways influenced by methylation dynamics, facilitating rapid adaptation to stress conditions [66]. Comparable observations have been reported in wheat, where drought-tolerant cultivars exhibited reduced global methylation levels under stress together with increased ABA accumulation and activation of stress-related genes including *Phosphoenolpyruvate Carboxylase* (*PEPC*), *Glutathione S-Transferase* (*GST*), and *Beta-Glucosidase* (*BGlu*) [67]. In *Camellia sinensis*, epigenetic regulation has likewise been associated with enhanced ABA biosynthesis during dehydration through the upregulation of key genes such as *9-cis-Epoxycarotenoid Dioxygenase* (*CsNCED*) and members of the *Zeaxanthin Epoxidase* (*CsZEP*) family [68].

#### 3.1.2. Developmental and Physiological Adaptations

Beyond ABA-related pathways, DNA methylation also contributes to developmental and physiological adaptations associated with drought tolerance. Studies on *Arabidopsis thaliana cmt3* mutants, defective in CHG methylation maintenance, demonstrated reduced stomatal density and enhanced drought tolerance across generations, highlighting the importance of stable methylation contexts in regulating traits associated with water-use efficiency [69]. In mulberry, drought-induced methylation changes affect genes involved in hormone signalling, particularly auxin-related pathways, through the modulation of interactions between Aux/IAA proteins and auxin response factors (ARFs) [70]. Although these genome-wide methylation studies reveal strong associations between DMRs and stress-responsive genes, they do not by themselves demonstrate causal regulatory relationships. Functional validation through mutant analysis, targeted epigenome editing, or gene perturbation approaches remains essential. A similar integration between methylation and hormonal regulation has been reported in *Dendrobium officinale*, where methylation-related genes contain multiple hormone-responsive cis-elements, indicating extensive crosstalk between epigenetic and hormonal signalling networks.

#### 3.1.3. Genome-Wide Methylation Reprogramming

Beyond individual genes and signalling pathways, drought also induces extensive genome-wide methylation reprogramming. Studies in crop species such as rice and maize have identified thousands of differentially methylated regions (DMRs), particularly within promoter regions of stress-responsive genes, demonstrating the large-scale epigenetic changes that accompany water deficit [71,72,73]. In grafted systems, however, interpretation of these responses may be complicated by the movement of regulatory molecules. Although grafting is not a drought-specific process, it provides valuable evidence for the stability, transmission, and biological significance of epigenetic regulatory mechanisms, thereby contributing to the broader understanding of stress-associated epigenetic memory. For example, drought-associated methylation patterns can interact with mobile miRNAs, including miR399, miR395, and miR172, making it difficult to distinguish direct epigenetic inheritance from other forms of systemic regulation [74]. In addition, coordinated changes in DNA methylation and gene expression have been observed in *Populus × euramericana* under different water regimes, particularly in genes associated with ABA, salicylic acid, and ethylene signalling pathways [75].

#### 3.1.4. Hormonal Crosstalk Across Species

Besides regulating ABA-dependent responses, DNA methylation also coordinates multiple hormonal pathways involved in drought adaptation across diverse plant species. In barley, RNA-directed DNA methylation has been implicated in gene silencing under terminal drought stress, with increased promoter methylation of *CYTOKININ-OXIDASE 2.1* (*HvCKX2.1*) suggesting a direct connection between epigenetic regulation and cytokinin metabolism [76]. In poplar, downregulation of the chromatin remodeller DECREASE OF DNA METHYLATION 1 (DDM1) increased drought tolerance and was accompanied by altered methylation patterns in hormone-related genes as well as changes in hormonal balance, including elevated salicylic acid and reduced cytokinin levels [77]. Likewise, studies in rice have shown that methylation dynamics influence pathways mediated by ABA, auxin, gibberellins, jasmonates, cytokinins, and brassinosteroids, ultimately affecting stress-responsive gene expression [78].

#### 3.1.5. Cotton, Cadmium and Conserved Mechanisms

Similar observations have been reported in cotton, where methylation regulates hormonal pathways involving ethylene, auxin, gibberellins, and cytokinins, thereby contributing to drought tolerance [79]. Genome-wide analyses have additionally identified cytochrome P450 genes displaying stress-responsive methylation profiles, further supporting a close relationship between epigenetic regulation and hormone metabolism [80]. Similar interactions between DNA methylation and hormone-regulated developmental processes have also been described under cadmium stress. Although cadmium and drought represent distinct abiotic stresses, this example is included because it demonstrates conserved epigenetic regulatory mechanisms that are shared among different environmental stress responses. Altered methylation states have been associated with changes in auxin transport, cytokinin accumulation, stem cell niche maintenance, ROS homeostasis, meristem activity, gravitropic responses, and broader stress-responsive transcriptional and metabolic networks, further supporting the role of epigenetic regulation in coordinating plant developmental plasticity and environmental adaptation [81,82,83,84,85].

#### 3.1.6. Overall Synthesis

Collectively, these findings demonstrate that DNA methylation functions as a central regulatory interface between environmental stress perception and hormonal signalling. More generally, hormone-regulated developmental responses can be influenced by a variety of environmentally responsive metabolites that affect auxin-dependent growth processes, root development, and adaptive plasticity, further illustrating the complexity of plant regulatory networks [86,87]. Drought-induced methylation changes are often dynamic and context-dependent, allowing plants to balance regulatory stability with adaptive plasticity. By influencing the biosynthesis, signalling, and interaction of phytohormones such as ABA, auxin, ethylene, gibberellins, cytokinins, jasmonates, and salicylic acid, DNA methylation contributes to both immediate stress responses and longer-term adaptive processes. These molecular changes are frequently associated with physiological traits including reduced stomatal conductance and improved water-use efficiency, thereby linking epigenetic regulation to whole-plant drought tolerance [18]. Similar physiological responses, including stomatal regulation, osmotic adjustment, and improved seedling performance under drought, have also been comprehensively reviewed by Seymen et al. [88]. Within the conceptual framework proposed here, DNA methylation can therefore be viewed as a key regulatory layer that stabilises hormone-mediated responses while contributing to the establishment of drought-associated stress memory. This interplay between epigenetic regulation and hormonal signalling represents an important component of plant resilience and a promising target for crop improvement strategies aimed at enhancing drought tolerance [77].

### 3.2. Histone Modifications Coordinate Hormonal and Developmental Responses to Drought

#### 3.2.1. Histone Modifications Regulate ABA-Dependent Drought Responses

Histone modifications represent an important regulatory layer linking hormonal signalling pathways with plant responses to drought stress. Through changes in histone methylation, acetylation, and chromatin accessibility, plants can dynamically regulate stress-responsive genes and fine-tune hormonal networks involved in adaptation to water deficit. Increasing evidence indicates that these modifications contribute not only to immediate stress responses but also to longer-term regulatory adjustments associated with environmental adaptation. Among the hormonal pathways influenced by histone modifications, ABA signalling has received particular attention. In barley, genome-wide ChIP-seq analyses revealed drought-induced changes in histone H3 modifications, especially H3K4 trimethylation and H3K9 acetylation. Notably, H3K9 acetylation showed strong enrichment at genes associated with ABA signalling, including members of the protein phosphatase 2C (PP2C) family, highlighting the importance of chromatin regulation in ABA-mediated drought responses [89].

#### 3.2.2. Histone-Modifying Enzymes Coordinate Hormonal and Developmental Adaptation

Beyond chromatin-wide changes, several studies have demonstrated that histone acetylation and deacetylation directly modulate ABA-dependent transcriptional responses during drought stress. Changes in histone modifications at drought-responsive loci further suggested a connection between WHY1 activity and epigenetic regulation of stress-associated gene expression [90]. A similar relationship between histone modifications and ABA-dependent pathways has been reported in sea buckthorn seedlings. Drought-induced H3K9 acetylation positively regulates genes involved in ABA biosynthesis and signalling, including *PP2C2*, *Dehydration-Responsive Element-Binding Protein 2* (*DREB2*), members of the NAC transcription factor family, and *SNF1-Related Protein Kinase 2* (*SRK2*). These changes influence ABA synthesis and distribution and subsequently affect downstream regulators such as *ABA Responsive Element-Binding Factor 2* (*ABF2*) [91]. Likewise, a novel RPD3/HDA1-type histone deacetylase identified in poplar, 84KHDA903, displayed differential expression under drought and ABA treatments. Transgenic tobacco plants expressing this gene exhibited enhanced drought tolerance accompanied by increased expression of stress-responsive genes including *NtDREB4*, *NtDREB3*, and *NtLEA5*, supporting a role for histone deacetylation in ABA-associated stress regulation [92]. In *Arabidopsis thaliana*, the histone deacetylases HD2A and HD2B are also integrated into ABA-dependent drought responses. Mutants lacking these proteins displayed enhanced drought resistance, smaller stomatal apertures, elevated reactive oxygen species (ROS) levels, and increased expression of drought-responsive genes, indicating that ABA-mediated feedback regulation contributes to transcriptional control through histone deacetylation mechanisms [93].

#### 3.2.3. Histone-Modifying Enzymes in Different Species

Beyond individual signalling pathways, several histone-modifying enzymes have emerged as important regulators of drought adaptation. In wheat (*Triticum aestivum*), Jumonji C (JmjC) domain-containing proteins coordinate complex histone methylation dynamics associated with stress tolerance. Genes including *Tr-1B-JMJ2*, *Tr-1A-JMJ2*, *Tr-1B-JMJ3*, *Tr-1D-JMJ2*, *Tr-7A-JMJ1*, and *Tr-4B-JMJ1* have been identified as important components of these epigenetic regulatory networks [94]. Histone deacetylases represent another major class of enzymes involved in hormone-mediated stress adaptation. In tomato (*Solanum lycopersicum*), the broadly expressed histone deacetylase gene *SlHDA5* is induced by ABA and methyl jasmonate (MeJA), and its silencing reduces tolerance to both salt and drought stress. These observations underscore the contribution of histone acetylation dynamics to hormone-dependent stress responses [95]. A comparable role has been described for SlHDA3, another tomato histone deacetylase whose silencing decreases drought and salt tolerance. In this system, ABA and gibberellic acid (GA3) influence stress adaptation, whereas indole-3-acetic acid (IAA) and salicylic acid (SA) participate in growth and defense responses, all of which are modulated through histone-dependent regulatory pathways [96].

#### 3.2.4. Developmental Responses and Conclusion

Histone modifications also contribute to developmental and long-term responses associated with environmental adaptation. In tomato, self-grafting induces extensive epigenetic reprogramming involving both histone and DNA modifications. Significant changes in histone H3K4 and H3K27 trimethylation, together with altered DNA methylation patterns, were associated with persistent effects on hormone-related gene expression, chromosomal organization, metabolic pathways, and stress-responsive processes [62]. Similarly, drought-induced early flowering in Chinese cabbage is regulated through interactions between histone modifications and hormonal signalling pathways. The histone H4 protein BrHIS4.A04 influences ABA-related responses and photoperiodic flowering genes, thereby linking chromatin regulation with developmental adaptation under water deficit conditions [97].

Collectively, these studies demonstrate that histone modifications function as dynamic regulators of hormonal signalling during drought stress. Through the coordinated action of histone methylation and acetylation pathways, plants can modulate ABA-dependent responses, regulate stress-responsive gene expression, and integrate developmental programmes with environmental adaptation. The principal hormonal pathways influenced by epigenetic regulation during drought stress are summarized in Table 2. Within the conceptual framework proposed in this review, histone modifications represent key components of the feedback loops connecting hormonal signalling, transcriptional regulation, and epigenetic memory, enabling flexible yet coordinated responses to drought stress.

### 3.3. MicroRNA-Mediated Regulation of Hormonal Signalling Under Drought Stress

miRNAs are important regulators of plant adaptation to drought, acting through the modulation of genes involved in hormone biosynthesis, perception, and signalling [104,105]. Their ability to coordinate multiple regulatory pathways places them at the centre of the molecular networks underlying drought acclimation. The development of high-throughput sequencing technologies has enabled the identification of large numbers of drought-responsive miRNAs across plant species. These studies have revealed extensive spatial and temporal variation in miRNA expression patterns, indicating that their responses are highly context-dependent and tightly linked to developmental stage and tissue type [104,106,107,108]. A major aspect of miRNA-mediated regulation concerns hormone-related pathways. In *Dendrobium huoshanense*, drought stress alters the expression of 211 miRNAs, including miR156, miR157d, and miR160a-5p, which are associated with auxin and cytokinin signalling and contribute to drought adaptation [100]. Likewise, in tomato, 699 miRNAs were identified during breeding for drought tolerance, with miR160, miR165, miR166, miR171, miR398, miR408, miR827, miR9472, miR9476, and miR9552 participating in hormone-mediated regulatory networks linked to both drought responses and tissue development [99]. Evidence from rice further highlights the broad regulatory influence of miRNAs. Chen and Li [101] identified 13 miRNAs targeting 58 mRNAs involved in hormone signalling, metabolism, and antioxidant defence pathways. Similarly, early studies based on partial root zone drying identified novel drought-responsive miRNAs whose expression patterns reflected the interaction between hormonal signalling and phosphorus homeostasis under changing water conditions [109]. The influence of miRNAs extends beyond classical hormone pathways. In maize roots, drought-induced morphological, physiological, and transcriptomic changes were accompanied by alterations in miRNA expression, revealing a close relationship between hormonal regulation and stress-responsive gene networks [110]. Comparable findings were reported in sweet potato, where analyses conducted under elevated CO_2_ and drought conditions revealed interactions among miRNAs, transcription factors, hormone regulators, and carbon metabolism pathways [103]. In *Camellia oleifera*, integrated mRNA-seq and miRNA-seq analyses identified drought-responsive genes associated with photosynthesis, chlorophyll metabolism, circadian rhythm, and hormone signalling, underscoring the central role of miRNAs in drought tolerance [111]. The contribution of miRNAs to drought adaptation has also been demonstrated in perennial species. In peach and almond, miR156, miR159, miR160, miR167, miR171, miR172, miR398, miR403, miR408, miR842, and miR2275 were associated with dehydration responses, while promoter analyses revealed the presence of hormone-responsive elements linked to their regulation [102]. In *Paulownia* “yuza 1”, Deng et al. [112] identified 107 miRNAs and 42 putative target genes related to drought adaptation, including functions associated with hormone signalling, osmotic adjustment, and photosynthesis. Additional regulatory complexity is provided by interactions between different classes of non-coding RNAs. In rice, Yang et al. [113] described a ceRNA network in which lncRNAs interact with miRNAs to regulate hormone signalling, chlorophyll biosynthesis, and protein production under drought stress. Among these interactions, MSTRG.28732.3 was proposed to act through miR171 and genes involved in chlorophyll biosynthesis, including *Os02g0662700*, *Os02g0663100*, and *Os06g0105350*. Taken together, these findings demonstrate that miRNAs act as central regulators of hormonal signalling pathways during drought stress. Beyond stress responses, miRNA-mediated regulation is also involved in developmental processes, including fruit maturation and ripening, further supporting the broad regulatory functions of small RNAs in plant growth and environmental adaptation [114]. Although these developmental processes are not directly related to drought stress, they illustrate conserved regulatory mechanisms that are also recruited during abiotic stress responses, highlighting the broad functional roles of miRNAs across different biological contexts.

By influencing hormone-related genes and interconnected metabolic processes, they contribute to the regulation of photosynthesis, osmotic balance, growth, and stress adaptation. Within the proposed framework, miRNAs represent key regulatory nodes linking signalling pathways with transcriptional and epigenetic regulation, thereby contributing to both rapid and sustained adaptive responses to drought.

### 3.4. Epi-miRNAs as Integrators of Epigenetic and Hormonal Responses

Among the diverse classes of drought-responsive miRNAs, increasing attention has been directed toward miRNAs that have been proposed to be under epigenetic regulation, here referred to as putative epi-miRNAs. It is important to distinguish experimentally validated epi-miRNAs, whose expression has been directly demonstrated to be regulated by epigenetic mechanisms, from drought-responsive miRNAs whose expression changes during stress but for which direct evidence of epigenetic regulation is still lacking. Unless otherwise stated, the examples discussed below should therefore be interpreted primarily as drought-responsive miRNAs with potential epigenetic regulation.

These molecules represent an additional layer of regulatory control, integrating post-transcriptional gene regulation with epigenetic mechanisms involved in plant stress adaptation [115,116]. Besides regulating target mRNAs, several miRNAs indirectly contribute to chromatin remodelling through interactions with the RNA-directed DNA methylation (RdDM) pathway. These interactions influence the recruitment of DNA methyltransferases and chromatin-associated proteins, thereby contributing to locus-specific DNA methylation and transcriptional gene silencing. Consequently, miRNA-mediated regulation represents an additional mechanistic link between post-transcriptional regulation and epigenetic control during drought responses. The significance of epi-miRNAs lies in their ability to participate in reciprocal interactions between small RNA pathways and epigenetic regulation. The interplay between miRNAs and epigenetic modifications contributes to the fine-tuning of stress-responsive gene expression, influencing chromatin accessibility and regulatory activity during drought conditions [13]. Through the regulation of DNA methyltransferases and histone-modifying enzymes, epi-miRNAs may affect chromatin organization and thereby modulate plant responses to environmental stress. Evidence for the involvement of drought-responsive miRNAs and putative epi-miRNAs in drought adaptation has been reported across several plant species. In *Solanum lycopersicum*, drought-responsive miRNAs such as miR160, miR166, and miR398 target genes associated with dehydration responses and stress adaptation pathways. These miRNAs regulate transcription factors and proteins, including dehydration-responsive proteins and glycosyltransferases, that contribute to adaptation under water-deficit conditions [99]. A broader picture emerged from the work of Chakraborty et al. [115], who identified 1002 miRNAs across multiple millet species, including 215 miRNAs targeting 155 major drought-responsive genes. These findings emphasize the extensive involvement of miRNA-mediated regulation in drought tolerance and suggest considerable potential for the improvement of crop performance under water-limited environments. The functional relevance of epi-miRNAs is particularly evident in pathways associated with ABA. MiRNAs contribute to drought adaptation through the regulation of ABA biosynthesis and signalling [117]. In rice, Gao et al. [98] demonstrated that *OsbZIP86* plays a central role in drought-induced ABA accumulation. In the absence of miR2105, *OsbZIP86* is activated by *OsSAPK10*, resulting in increased *OsNCED3* expression and enhanced ABA production. Consequently, plants carrying miR2105 knockdown constructs or overexpressing *OsbZIP86* exhibited increased ABA accumulation, reduced water loss, enhanced stomatal closure, and improved drought tolerance. Conversely, overexpression of miR2105 and downregulation or knockout of *OsbZIP86* led to reduced ABA content and lower drought resilience. Although the importance of epi-miRNAs is increasingly recognized, relatively few have been experimentally validated to date. Consequently, current efforts are focused on identifying additional miRNAs under epigenetic control and clarifying their specific contributions to drought adaptation [118]. Overall, although only a limited number of miRNAs have been experimentally validated as epi-miRNAs, increasing evidence suggests that drought-responsive miRNAs interact closely with epigenetic pathways and may contribute to chromatin-based regulation. By connecting post-transcriptional regulation with epigenetic mechanisms, they provide a functional link between chromatin-based regulation and stress-responsive signalling pathways. Within the proposed conceptual framework, epi-miRNAs can therefore be viewed as integrative elements that bridge epigenetic regulation and hormonal signalling, contributing to both immediate adaptive responses and longer-term regulatory adjustments.

### 3.5. Epitranscriptomic Regulation in Plant Drought Responses

In addition to canonical epigenetic layers—DNA methylation, histone modifications, and small RNA pathways—plants also employ epitranscriptomic regulation, defined as chemical modifications of RNA that modulate gene expression at the post-transcriptional level [91,119,120,121]. This regulatory layer enables rapid and reversible adjustments of gene expression, making it particularly relevant under fluctuating environmental conditions such as drought stress. The plant epitranscriptome encompasses a wide range of RNA modifications (with more than 150 described across organisms), among which N6-methyladenosine (m^6^A) and 5-methylcytosine (m^5^C) are the most extensively studied in mRNA [28,119,120,122]. Among these, m^6^A is considered the most abundant internal modification of eukaryotic mRNA and serves as a model for understanding epitranscriptomic regulation in plants [28,120,123].

Mechanistically, m^6^A operates through a “writer–reader–eraser” system involving methyltransferases, RNA-binding proteins (often YTH-domain proteins), and demethylases, which collectively regulate transcript stability, splicing, export, and translation efficiency [119,120,121,123]. Functionally, these modifications influence RNA fate by modulating RNA–protein interactions and local RNA structure, thereby affecting gene expression outputs. In many cases, m^6^A is enriched near stop codons and in 3′ UTR regions, suggesting that its positional distribution contributes to its regulatory function [28,121,123].

With respect to drought adaptation, epitranscriptomic regulation provides a mechanism for rapid modulation of stress-responsive transcripts. Available studies indicate that RNA methylation pathways respond dynamically to abiotic stress and can influence transcript stability and translation efficiency [120,121,122]. In particular, m^6^A deposition on stress-related transcripts has been associated with enhanced stability, reduced RNA secondary structure, and increased protein production, supporting its role in osmotic stress responses, a key component of drought physiology [91]. Although direct drought-specific evidence remains limited and context-dependent, these findings support the view that epitranscriptomic regulation contributes to transcriptome plasticity under water deficit conditions [91,120].

In addition to m^6^A, other RNA modifications such as m^5^C, pseudouridine (Ψ), and 2′-O-methylation (Nm) are increasingly recognized, although their functional roles in plants remain less well characterized due to technical limitations in detection and quantification [28,119]. Nonetheless, these modifications are thought to act as regulatory switches affecting RNA processing, translation, and decay, processes that are particularly important under rapidly changing environmental conditions.

Within the conceptual framework proposed in this review, epitranscriptomic regulation represents a highly dynamic layer that complements chromatin-based epigenetic mechanisms. Unlike DNA methylation or histone modifications, RNA modifications are generally not considered stably heritable, as they are lost upon transcript degradation. Consequently, their contribution to stress “memory” is best interpreted as transient, operating at the level of RNA persistence rather than long-term epigenetic inheritance [124,125].

Importantly, epitranscriptomic regulation may interact with both hormonal signalling and miRNA-mediated pathways. For instance, regulatory regions of m^6^A-related genes often contain hormone-responsive cis-elements, suggesting that hormonal signals may modulate the epitranscriptomic machinery [126]. In addition, bidirectional interactions between m^6^A and miRNA pathways have been proposed, whereby RNA methylation may influence miRNA binding and, conversely, miRNAs may affect m^6^A deposition, although much of this evidence derives from non-plant systems and requires further validation in plant drought contexts [124,127].

Overall, epitranscriptomic regulation can be integrated into drought-response models as a rapid and flexible post-transcriptional control layer that interacts with hormonal and small RNA pathways. While its mechanistic role in drought adaptation is still being elucidated, current evidence supports its contribution to fine-tuning gene expression and enhancing plant responsiveness to water deficit conditions [28,120,122,124,125].

## 4. Technological Advances in Studying Epigenetic Modifications

Investigating epigenetic modifications has become essential for understanding the complex regulatory mechanisms controlling gene expression, particularly in the context of plant responses to environmental stresses such as drought. Recent technological innovations have transformed the study of epigenetics, offering powerful tools to dissect these molecular processes [128]. This section highlights two major advancements: high-throughput approaches for profiling epigenetic changes and the use of CRISPR-Cas9 for engineering epigenetic regulation to improve drought tolerance.

### 4.1. Profiling Drought-Induced Epigenetic Landscapes

The development of high-throughput technologies has transformed the study of plant epigenetics, enabling genome-wide characterization of DNA methylation, histone modifications, chromatin accessibility, and regulatory elements involved in stress adaptation. Combined with next-generation sequencing, these approaches provide unprecedented insights into the molecular mechanisms underlying drought responses and generate large-scale datasets that support the identification of stress-responsive regulatory networks [107,129].

Among these approaches, DNA methylation profiling has become a fundamental tool for investigating drought-associated epigenetic regulation. Bisulfite sequencing remains the gold standard for detecting 5-methylcytosine at single-nucleotide resolution, providing detailed maps of methylation patterns across plant genomes [130]. Applications of methylation-based approaches have revealed important mechanisms associated with drought adaptation. For example, Shaik and Ramakrishna [131] used methylcytosine immunoprecipitation coupled with sequencing to analyse drought-responsive genes in rice, uncovering complex interactions among DNA methylation, miRNA regulation, and gene expression, particularly in chromatin-remodelling genes. Similarly, Garg et al. [132] employed high-throughput sequencing to generate single-base-resolution methylation maps in three rice cultivars, identifying differentially methylated regions (DMRs) associated with genes involved in drought responses. High-throughput sequencing has also facilitated the study of epimutations. Zheng et al. [133] developed rice lines that accumulated epimutations over successive generations of drought exposure, demonstrating that these modifications occur non-randomly and contribute to drought adaptation. Likewise, Wang et al. [71] showed that increased DNA methylation in maize roots is associated with water stress and drought tolerance. Together, these studies highlight the value of methylation profiling for identifying drought-responsive epigenetic signatures and potential markers for crop improvement.

Genome-wide analyses of histone modifications have similarly benefited from advances in sequencing technologies. Chromatin immunoprecipitation sequencing (ChIP-seq) enables the mapping of specific histone marks across the genome and has substantially improved our understanding of chromatin-mediated stress regulation [134]. In barley, Ost et al. [89] used ChIP-seq to identify drought-induced changes in H3K9 acetylation and H3K4 trimethylation, revealing strong associations with ABA signalling pathways and activation of drought-responsive genes. Song et al. [135] reported positive correlations between H3K9ac enrichment and drought-responsive gene expression in *Brachypodium distachyon*, while Dasgupta et al. [136] demonstrated links between H3K27 modifications and stress-responsive gene activation in rice. Furthermore, Zong et al. [137] identified interactions between H3K4me3 and the transcription factor OsbZIP23 in regulating drought-responsive genes. More recently, Zhao et al. [138] highlighted the role of the histone demethylase JMJ710 in rice, showing that its overexpression increases drought sensitivity through altered expression of stress-related genes. Collectively, these studies demonstrate the utility of ChIP-seq-based approaches for dissecting chromatin-level regulation of drought responses.

In addition to DNA methylation and histone profiling, methods that assess chromatin accessibility have provided new perspectives on gene regulation under water deficit conditions. Assays such as ATAC-seq (Assay for Transposase-Accessible Chromatin with sequencing) generate genome-wide maps of accessible chromatin regions and facilitate the identification of regulatory elements involved in stress adaptation [139]. Using ATAC-seq, Mladenov et al. (2023) [140] performed the first epigenetic analysis of the desiccation-tolerant species *Haberlea rhodopensis*, while Yang et al. [141] demonstrated that OsNMCP1 influences chromatin accessibility at drought-responsive loci in rice, affecting root development and drought tolerance. Comparative ATAC-seq studies across cultivars and species offer additional opportunities to identify conserved and species-specific regulatory mechanisms associated with drought resilience.

The growing volume of epigenomic information generated by these technologies has stimulated the development of computational approaches for data integration and interpretation. Advances in computational epigenetics have improved the analysis of large-scale datasets and facilitated the construction of comprehensive epigenetic maps that capture the complexity of drought-responsive regulatory networks [128,142,143,144]. Integrating methylation, chromatin accessibility, histone modification, and transcriptomic datasets is increasingly enabling a systems-level understanding of plant adaptation to drought stress and accelerating the identification of candidate genes and regulatory pathways relevant for crop improvement. Recent studies further demonstrate that combining epigenomic datasets with artificial intelligence and high-throughput phenotyping pipelines can substantially improve the identification of stress-responsive biomarkers and predictive models for crop improvement [145].

### 4.2. Functional Dissection and Epigenome Engineering Approaches

The CRISPR-Cas9 system has transformed plant biology by enabling precise and efficient genome editing of specific DNA sequences [146].

Conventional CRISPR/Cas9-mediated genome editing has become an essential tool for investigating the functions of epigenetic regulators involved in plant stress responses. By generating knockout or knock-in mutants, researchers can determine the biological roles of genes controlling DNA methylation, histone modifications, and chromatin remodelling. For example, targeted disruption of the chromatin remodeller DECREASE IN DNA METHYLATION1 (DDM1) in tomato (*Solanum lycopersicum*) has improved our understanding of methylation-dependent genome stability and transposon silencing [147]. Similarly, CRISPR/Cas9-mediated knockout of the histone deacetylase ZmHDT103 in maize demonstrated that this regulator contributes to drought tolerance, with mutant plants exhibiting enhanced resilience under water-deficit conditions [148]. These studies provide functional evidence linking epigenetic regulators to drought-responsive phenotypes but do not constitute targeted epigenome editing, as the underlying DNA sequence is permanently modified.

In contrast, CRISPR-based epigenome editing relies on catalytically inactive Cas proteins (dCas9) fused to epigenetic effector domains, allowing locus-specific modification of DNA methylation or histone marks without altering the underlying DNA sequence [149,150,151]. For example, the catalytic domain of the *Arabidopsis* ROS1 5-methylcytosine DNA glycosylase has been fused to dCas9 to reactivate genes silenced by DNA methylation [152]. Likewise, fusion of dCas9 with histone-modifying enzymes enables targeted changes in chromatin state, providing a powerful approach to directly test causal relationships between specific epigenetic modifications and drought-responsive phenotypes [153].

Together, conventional genome editing and targeted epigenome editing provide complementary strategies for investigating plant drought adaptation. While CRISPR/Cas9 knockout approaches reveal the biological functions of epigenetic regulators, dCas9-based epigenome editing enables direct manipulation of chromatin states at specific loci without modifying the genomic sequence. The combination of these approaches will be instrumental in translating epigenetic discoveries into innovative strategies for crop improvement.

The principal technologies currently used to investigate and manipulate drought-associated epigenetic regulation are summarized in Table 3.

### 4.3. Integration and Future Applications of Epigenomic Technologies

Recent technological advances have substantially expanded our understanding of the epigenetic mechanisms underlying plant responses to drought stress. High-throughput approaches, including bisulfite sequencing, ChIP-seq, and ATAC-seq, have enabled genome-wide characterization of DNA methylation patterns, histone modifications, and chromatin accessibility, providing unprecedented insights into the regulatory networks that govern stress-responsive gene expression. These technologies have revealed the complexity of epigenetic regulation and facilitated the identification of molecular pathways associated with drought adaptation.

At the same time, the emergence of CRISPR-based genome and epigenome engineering has provided powerful tools for investigating the functional significance of specific epigenetic modifications. Targeted manipulation of DNA methylation and histone marks has improved our ability to establish causal relationships between epigenetic regulation and stress-responsive phenotypes, while also opening new opportunities for the development of crops with enhanced drought tolerance.

Together, these complementary approaches are accelerating the transition from descriptive epigenetic studies to functional and translational research. The integration of high-resolution epigenomic profiling with targeted epigenome engineering is expected to facilitate the identification of key regulatory loci and accelerate the development of innovative strategies for crop improvement under increasingly challenging environmental conditions.

## 5. Challenges and Open Questions

Despite major progress in profiling drought-responsive epigenetic layers, a key unresolved issue is causality: many drought-associated changes in DNA/RNA marks are identified through genome-/transcriptome-wide mapping, but their mechanistic contribution to drought phenotypes often remains inferred rather than directly demonstrated, making functional validation essential [119,120,121,122]. This limitation is repeatedly emphasized in epigenome/epitranscriptome-for-breeding perspectives, which note that practical exploitation requires a deeper mechanistic understanding of how specific marks and their regulatory machineries influence RNA fate and downstream traits under stress [119,120,122]. Recent progress in programmable RNA editing (e.g., CRISPR/dCas13-based targeted installation/removal of m^6^A) provides a concrete route to test causality at the level of individual transcripts and assess phenotypic impact under drought, but these approaches are still emerging and require broader validation across crops and environments [28,119,122].

A second open question concerns the stability and (trans)generational persistence of “epigenetic memory.” Multiple reviews highlight that stress can induce epigenetic reprogramming that may contribute to memory-like effects, yet the duration, reversibility, and heritability of these states vary across contexts and remain insufficiently predictable for routine deployment in crop improvement [120,156,157]. In climate-change syntheses, drought is explicitly discussed as a driver of DNA methylation changes that may sometimes be retained after stress relief, supporting the plausibility of persistence while also underscoring context dependence [156]. More broadly, reviews of stress-induced epigenetic/metabolic regulation emphasize outstanding gaps regarding the temporal dynamics, tissue specificity, and long-term stability of epigenetic marks in crops—parameters that are decisive for whether a “memory” state can be harnessed safely and reproducibly in agriculture [120,123,157].

A third bottleneck is translation to breeding and field performance. Crop-improvement reviews argue that epigenetic and epitranscriptomic diversity could expand the breeding toolbox, but they also stress that translation requires (i) robust mechanistic links between marks and traits and (ii) validation in agronomically relevant settings, including field evaluation [119,120,121]. In rice-focused epitranscriptomic syntheses, m^6^A dynamics are proposed as a promising lever to tune drought responses, yet the same analyses explicitly call for careful functional validation and field evaluation before broad crop deployment [121]. Technically, these goals are constrained by continuing challenges in epitranscriptome measurement—particularly achieving high-resolution, quantitative, and comparable detection of multiple RNA marks across tissues and stress regimes—although third-generation and direct RNA sequencing approaches are expanding what is currently measurable [119,121,126].

Finally, an important (and sometimes under-tested) consideration is the potential for fitness or yield trade-offs. Stress responses are tightly linked to metabolic reprogramming (e.g., redirected carbon/nitrogen fluxes toward protective metabolites), which is itself under epigenetic control, implying that drought-adaptive states may carry opportunity costs under non-stress conditions [157]. In parallel, plant m^6^A syntheses explicitly frame epitranscriptome engineering as a means to fine-tune trade-offs via more precise regulation of gene expression, highlighting that understanding when stress-enhancing regulation becomes growth-limiting remains an open question rather than a settled outcome [119,122,124].

Despite significant advances, several key questions remain open, defining priorities for future research. A central challenge is to distinguish causal regulatory mechanisms from downstream molecular signatures, particularly in the context of epigenetic and epitranscriptomic modifications. Establishing causality will require integrating functional approaches, such as targeted epigenome and RNA editing, to validate candidate regulatory elements across diverse genetic backgrounds [28,119,121,122].

Another critical issue concerns the persistence and stability of stress-induced memory. While epigenetic and epitranscriptomic modifications can contribute to adaptive responses, the extent to which these changes are maintained within an individual plant or transmitted across generations, and how environmental variability influences their stability, remains incompletely understood [120,122,123,156].

From an applied perspective, translating epigenetic knowledge into breeding strategies represents a major challenge. Developing field-relevant predictive models will require multi-environment validation and a better understanding of how regulatory mechanisms perform under realistic agricultural conditions [119,120,121].

Finally, the agronomic consequences of maintaining stress-responsive states must be carefully evaluated. While these mechanisms enhance drought tolerance, they may also involve fitness trade-offs that affect growth and productivity under non-stress conditions, highlighting the need to balance resilience with yield optimization [121,124,157].

## 6. Future Prospects

Future advances in plant drought biology will likely depend on the integration of multiple regulatory layers rather than on the study of individual mechanisms in isolation. A major challenge for future research will be the development of comprehensive models capable of linking epigenetic modifications, hormonal signalling pathways, non-coding RNAs, and epitranscriptomic regulation within unified drought-response networks. The increasing availability of multi-omics datasets offers unprecedented opportunities to address this challenge. Integrating epigenomic, transcriptomic, proteomic, and metabolomic information may facilitate the identification of key regulatory hubs controlling drought adaptation and improve our understanding of the complex interactions underlying stress-responsive phenotypes. Recent developments in genome and epigenome engineering further provide promising opportunities for translating fundamental discoveries into crop improvement strategies. Targeted manipulation of DNA methylation, histone modifications, and regulatory RNAs may enable the modification of specific drought-responsive pathways while minimizing undesired effects on plant growth and development. Another important research priority concerns the establishment and persistence of drought-associated stress memory. Although increasing evidence supports the existence of epigenetic memory mechanisms, important questions remain regarding their maintenance after stress recovery, their stability across generations, and their potential exploitation in breeding programmes. Future studies should also clarify the role of phytohormone–epigenetic crosstalk in coordinating drought responses. Expanding our understanding of how environmental and developmental signals converge to regulate gene expression may further improve the interpretation of plant adaptive responses under changing conditions [158]. Understanding how hormonal signals influence chromatin dynamics and how epigenetic states affect hormone sensitivity may reveal key regulatory nodes controlling plant adaptation to water deficit. Finally, expanding research on non-coding RNAs, epitranscriptomic regulation, and stress-associated traits such as root architecture, stomatal development, and water-use efficiency will provide additional insights into the mechanisms through which plants achieve drought resilience.

## 7. Conclusions

As highlighted by the conceptual framework proposed in this review, plant adaptation to drought emerges from the coordinated interaction of multiple regulatory layers, including chromatin-based epigenetic mechanisms, hormonal signalling pathways, and small RNA-mediated regulation.

DNA methylation and histone modifications play central roles in modulating gene expression, enabling both rapid physiological responses and the establishment of longer-term adaptive memory. These chromatin-level processes are tightly integrated with hormonal signalling networks, particularly those involving abscisic acid (ABA), ethylene, jasmonates, and salicylic acid, which collectively fine-tune plant responses to water deficit. In parallel, miRNAs act as key regulatory nodes linking transcriptional, post-transcriptional, and epigenetic processes, reinforcing the coordination between short-term responses and sustained adaptive strategies.

Emerging evidence further indicates that epitranscriptomic modifications, such as m^6^A RNA methylation, add an additional layer of regulation by dynamically modulating transcript stability and translation efficiency. Although their role in drought adaptation is still being elucidated, these mechanisms are likely to contribute to the fine-tuning of gene expression and to rapid, reversible stress responses.

Importantly, drought adaptation must also be considered in light of potential fitness trade-offs. While stress-induced regulatory states enhance survival under adverse conditions, they may impose metabolic and developmental costs when stress is absent, highlighting the need to balance resilience with growth and productivity.

Technological advances, including high-throughput epigenomic profiling and CRISPR-based epigenome and epitranscriptome editing, are rapidly expanding our ability to dissect causal relationships and to engineer stress-responsive regulatory networks. These tools provide unprecedented opportunities to translate mechanistic insights into practical applications for crop improvement.

Overall, integrating epigenetic, epitranscriptomic, hormonal, and small RNA-mediated regulation into a unified framework offers a powerful perspective on plant drought responses. Future research should focus on identifying causal regulatory elements, clarifying the stability and heritability of stress-induced modifications, and evaluating their impact under field conditions. Such efforts will be essential for developing resilient crops capable of sustaining productivity in the face of increasing environmental challenges. Bridging controlled experimental insights with field-level complexity will be critical to fully harness these regulatory mechanisms in real-world agricultural systems.

Together, these insights redefine plant drought adaptation as a multi-layered and dynamic regulatory process, where the integration of epigenetic, epitranscriptomic, and hormonal networks represents a key frontier for both fundamental research and sustainable crop improvement.

Looking ahead, translating these advances into practical breeding programmes will require the integration of multi-omics datasets, artificial intelligence-assisted predictive models, and functional validation through genome and epigenome editing. In addition, the identification and exploitation of naturally occurring epialleles and precision epigenetic engineering offer promising opportunities to develop climate-resilient crops with enhanced drought tolerance while minimizing potential trade-offs between stress adaptation, growth, and yield. Together, these approaches are expected to accelerate the implementation of epigenetic knowledge into next-generation breeding and biotechnological strategies for sustainable agriculture under changing climatic conditions.

## Figures and Tables

**Table 1:** Mechanisms involved in drought-associated epigenetic memory and inheritance.

Regulatory Mechanism	Molecular Components	Role in Drought Adaptation	Potential Persistence and Inheritance	References
DNA methylation	CG, CHG and CHH methylation; DNA methyltransferases	Regulation of stress-responsive gene expression and establishment of drought memory	May persist after stress exposure; transgenerational stability appears species- and context-dependent	[31,41,42,43]
Histone modifications	H3K4me3, H3K27me3 and other chromatin marks	Modulation of chromatin accessibility and transcriptional memory	May contribute to maintenance of stress-responsive states following drought exposure	[4,13,44]
Small interfering RNAs (siRNAs)	RdDM-associated siRNAs	Guidance of DNA methylation and transcriptional gene silencing	Potential transmission through reproductive tissues	[45,46,47,48]
Long non-coding RNAs (lncRNAs)	Regulatory lncRNAs associated with chromatin complexes	Recruitment of chromatin-modifying complexes and stabilization of gene expression states	Potential contribution to heritable epigenetic regulation	[39,40,50,51,52]
Chromatin remodelling	SWR1 complex, SWC6, SUF3, PIE1	Regulation of nucleosome composition and stress-responsive transcription	May influence maintenance and resetting of epigenetic states	[39,40]
Epigenetic resetting mechanisms	HDMs, HDACs, DUBs, phosphatases	Removal or modification of epigenetic marks during recovery and development	Prevents maladaptive persistence of stress-induced states	[34,35,36,37,38]

**Table 2:** Hormonal pathways influenced by epigenetic regulation during drought stress.

Hormonal Pathway	Epigenetic Mechanism	Representative Examples	Physiological or Developmental Outcome	References
Abscisic acid (ABA)	DNA methylation, histone modifications, miRNA regulation	*vp10* maize mutant, citrus, wheat, tea plant, barley, sea buckthorn, rice miR2105–OsbZIP86 module	Stomatal regulation, ABA accumulation, activation of stress-responsive genes, enhanced drought tolerance	[64,65,66,67,68,89,91,98]
Auxin	DNA methylation and miRNA-mediated regulation	Mulberry; *Dendrobium huoshanense*; tomato drought-responsive miRNAs	Root development, growth adjustment and drought adaptation	[70,99,100]
Cytokinins	DNA methylation and miRNA-mediated regulation	Barley (HvCKX2.1); poplar; *Dendrobium huoshanense*	Growth regulation and adaptation to water deficit	[76,77,100]
Ethylene	DNA methylation-mediated regulation	Cotton; *Populus × euramericana*	Modulation of stress-responsive pathways	[75,79]
Gibberellins (GA)	DNA methylation and histone modifications	Rice; cotton; tomato (SlHDA3)	Coordination of growth and drought responses	[78,79,96]
Jasmonates (JA)	DNA methylation and histone modification pathways	Rice; tomato (SlHDA5)	Regulation of stress signalling and adaptive responses	[78,95]
Salicylic acid (SA)	DNA methylation and histone-associated regulation	Poplar; tomato	Hormonal balance and stress acclimation	[75,77,96]
Multiple hormonal pathways	DNA methylation and miRNA-mediated regulation	Rice, cotton, maize, peach, almond, sweet potato	Coordination of drought-responsive transcriptional and physiological processes	[78,79,101,102,103]

**Table 3:** Technologies used to investigate and manipulate drought-associated epigenetic regulation.

Technology	Epigenetic Feature Analysed or Manipulated	Main Application	Representative Findings	References
CRISPR/Cas9 genome editing	Functional analysis of epigenetic regulators	Gene knockout/knock-in for functional validation	Validation of the roles of candidate epigenetic regulators in drought tolerance through targeted gene disruption or insertion.	[146]
CRISPR/dCas9 epigenome editing	DNA methylation and histone modifications	Targeted epigenetic engineering	Programmable editing of DNA methylation and chromatin states enables targeted modulation of stress-responsive gene expression.	[149,150]
Bisulfite sequencing	DNA methylation	Identification of differentially methylated regions (DMRs)	Genome-wide methylation profiling associated with drought adaptation	[130,132]
MeDIP-seq	DNA methylation	Characterization of methylation patterns in stress-responsive genes	Links between DNA methylation, miRNAs and drought-responsive gene expression	[154]
Small RNA sequencing	miRNAs	Discovery of drought-responsive regulatory RNAs	Identification of known and novel drought-associated miRNAs	[155]
High-throughput sequencing (HTS)	Genome-wide epigenetic variation	Detection of epimutations and stress-associated genomic changes	Identification of drought-induced epimutations and regulatory networks	[133]
ChIP-seq	Histone modifications	Mapping of chromatin-associated regulatory marks	Identification of drought-responsive histone acetylation and methylation patterns	[89,134,135,136,137]
ATAC-seq	Chromatin accessibility	Identification of regulatory regions and accessible chromatin	Characterization of chromatin accessibility associated with drought adaptation	[139,140,141]
Computational epigenomics	Multi-omics integration	Reconstruction of regulatory networks	Generation of integrated epigenetic maps associated with drought responses	[128,142,143,144]
CRISPR/dCas9 epigenome editing	DNA methylation and histone modifications	Functional validation and targeted epigenetic engineering	Targeted manipulation of epigenetic regulators involved in drought tolerance	[149,150,151,152,153]

## Data Availability

No new data were created or analyzed in this study. Data sharing is not applicable to this article.

## References

[1] Kumar M., Kumar Patel M., Kumar N., Bajpai A.B., Siddique K.H.M. (2021). Metabolomics and Molecular Approaches Reveal Drought Stress Tolerance in Plants. Int. J. Mol. Sci..

[2] Yang X., Lu M., Wang Y., Wang Y., Liu Z., Chen S. (2021). Response Mechanism of Plants to Drought Stress. Horticulturae.

[3] Sati D., Pande V., Pandey S.C., Samant M. (2023). Recent Advances in PGPR and Molecular Mechanisms Involved in Drought Stress Resistance. J. Soil Sci. Plant Nutr..

[4] Godwin J., Farrona S., Spillane C., McKeown P. (2020). Plant Epigenetic Stress Memory Induced by Drought: A Physiological and Molecular Perspective. Plant Epigenetics and Epigenomics: Methods and Protocols.

[5] Chung S., Kwon C., Lee J.-H. (2022). Epigenetic Control of Abiotic Stress Signaling in Plants. Genes Genom..

[6] Talarico E., Zambelli A., Araniti F., Greco E., Chiappetta A., Bruno L. (2024). Unravelling the Epigenetic Code: DNA Methylation in Plants and Its Role in Stress Response. Epigenomes.

[7] Temel A., Janack B., Humbeck K. (2017). Drought Stress-Related Physiological Changes and Histone Modifications in Barley Primary Leaves at HSP17 Gene. Agronomy.

[8] Czajka K., Mehes-Smith M., Nkongolo K. (2022). DNA Methylation and Histone Modifications Induced by Abiotic Stressors in Plants. Genes Genom..

[9] Kim J.-M., Sasaki T., Ueda M., Sako K., Seki M. (2015). Chromatin Changes in Response to Drought, Salinity, Heat, and Cold Stresses in Plants. Front. Plant Sci..

[10] Daszkowska-Golec A. (2016). The Role of Abscisic Acid in Drought Stress: How ABA Helps Plants to Cope with Drought Stress. Drought Stress Tolerance in Plants.

[11] Muhammad Aslam M., Waseem M., Jakada B.H., Okal E.J., Lei Z., Saqib H.S.A., Yuan W., Xu W., Zhang Q. (2022). Mechanisms of Abscisic Acid-Mediated Drought Stress Responses in Plants. Int. J. Mol. Sci..

[12] Kinoshita T., Seki M. (2014). Epigenetic Memory for Stress Response and Adaptation in Plants. Plant Cell Physiol..

[13] Sun C., Ali K., Yan K., Fiaz S., Dormatey R., Bi Z., Bai J. (2021). Exploration of Epigenetics for Improvement of Drought and Other Stress Resistance in Crops: A Review. Plants.

[14] Sadhukhan A., Prasad S.S., Mitra J., Siddiqui N., Sahoo L., Kobayashi Y., Koyama H. (2022). How Do Plants Remember Drought?. Planta.

[15] Kumar S., Seem K., Mohapatra T. (2023). Biochemical and Epigenetic Modulations under Drought: Remembering the Stress Tolerance Mechanism in Rice. Life.

[16] Begcy K., Dresselhaus T. (2018). Epigenetic Responses to Abiotic Stresses during Reproductive Development in Cereals. Plant Reprod..

[17] Karalija E., Ibragić S., Dahija S., Šamec D. (2025). Transgenerational Memory of Phenotypic Traits in Plants: Epigenetic Regulation of Growth, Hormonal Balance, and Stress Adaptation. Curr. Issues Mol. Biol..

[18] Kaya C., Uğurlar F., Adamakis I.-D.S. (2024). Epigenetic Modifications of Hormonal Signaling Pathways in Plant Drought Response and Tolerance for Sustainable Food Security. Int. J. Mol. Sci..

[19] Nguyen N.H., Vu N.T., Cheong J.-J. (2022). Transcriptional Stress Memory and Transgenerational Inheritance of Drought Tolerance in Plants. Int. J. Mol. Sci..

[20] Prova A., Sultan M.S., Kimatu J.N. (2022). Techniques against Distinct Abiotic Stress of Rice. Advances in Plant Defense Mechanisms.

[21] Matsui A., Nguyen A.H., Nakaminami K., Seki M. (2013). Arabidopsis Non-Coding RNA Regulation in Abiotic Stress Responses. Int. J. Mol. Sci..

[22] Bhadouriya S.L., Mehrotra S., Basantani M.K., Loake G.J., Mehrotra R. (2021). Role of Chromatin Architecture in Plant Stress Responses: An Update. Front. Plant Sci..

[23] Chinnusamy V., Gong Z., Zhu J.-K. (2008). Abscisic Acid-Mediated Epigenetic Processes in Plant Development and Stress Responses. J. Integr. Plant Biol..

[24] Dhar M.K., Vishal P., Sharma R., Kaul S. (2014). Epigenetic Dynamics: Role of Epimarks and Underlying Machinery in Plants Exposed to Abiotic Stress. Int. J. Genom..

[25] Kong L., Liu Y., Wang X., Chang C. (2020). Insight into the Role of Epigenetic Processes in Abiotic and Biotic Stress Response in Wheat and Barley. Int. J. Mol. Sci..

[26] Grgić M., Vitko S., Drmić J., Leljak-Levanić D. (2025). Connecting the Dots: Epigenetics, ABA, and Plant Stress Tolerance. Acta Bot. Croat..

[27] Kaya C., Adamakis I.-D.S. (2025). Redox-Epigenetic Crosstalk in Plant Stress Responses: The Roles of Reactive Oxygen and Nitrogen Species in Modulating Chromatin Dynamics. Int. J. Mol. Sci..

[28] Miryeganeh M. (2021). Plants’ Epigenetic Mechanisms and Abiotic Stress. Genes.

[29] Duarte-Aké F., Us-Camas R., De-la-Peña C. (2023). Epigenetic Regulation in Heterosis and Environmental Stress: The Challenge of Producing Hybrid Epigenomes to Face Climate Change. Epigenomes.

[30] Sun R.-Z., Liu J., Wang Y.-Y., Deng X. (2021). DNA Methylation-Mediated Modulation of Rapid Desiccation Tolerance Acquisition and Dehydration Stress Memory in the Resurrection Plant *Boea hygrometrica*. PLoS Genet..

[31] Cao X., Aufsatz W., Zilberman D., Mette M.F., Huang M.S., Matzke M., Jacobsen S.E. (2003). Role of the DRM and CMT3 Methyltransferases in RNA-Directed DNA Methylation. Curr. Biol..

[32] Tahir M.S., Karagiannis J., Tian L. (2022). HD2A and HD2C Co-Regulate Drought Stress Response by Modulating Stomatal Closure and Root Growth in *Arabidopsis*. Front. Plant Sci..

[33] Forestan C., Farinati S., Zambelli F., Pavesi G., Rossi V., Varotto S. (2020). Epigenetic Signatures of Stress Adaptation and Flowering Regulation in Response to Extended Drought and Recovery in *Zea mays*. Plant Cell Environ..

[34] Luo M., Hung F.-Y., Yang S., Liu X., Wu K. (2014). Histone Lysine Demethylases and Their Functions in Plants. Plant Mol. Biol. Rep..

[35] Singh A., Pandey G.K. (2012). Protein Phosphatases: A Genomic Outlook to Understand Their Function in Plants. J. Plant Biochem. Biotechnol..

[36] Luo R., Yang K., Xiao W. (2023). Plant Deubiquitinases: From Structure and Activity to Biological Functions. Plant Cell Rep..

[37] Ma X., Lv S., Zhang C., Yang C. (2013). Histone Deacetylases and Their Functions in Plants. Plant Cell Rep..

[38] Li S., He X., Gao Y., Zhou C., Chiang V.L., Li W. (2021). Histone Acetylation Changes in Plant Response to Drought Stress. Genes.

[39] Choi K., Park C., Lee J., Oh M., Noh B., Lee I. (2007). *Arabidopsis* Homologs of Components of the SWR1 Complex Regulate Flowering and Plant Development. Development.

[40] Aslam M., Fakher B., Jakada B.H., Cao S., Qin Y. (2019). SWR1 Chromatin Remodeling Complex: A Key Transcriptional Regulator in Plants. Cells.

[41] Hauser M.-T., Aufsatz W., Jonak C., Luschnig C. (2011). Transgenerational Epigenetic Inheritance in Plants. Biochim. Biophys. Acta BBA-Gene Regul. Mech..

[42] Herman J.J., Sultan S.E. (2016). DNA Methylation Mediates Genetic Variation for Adaptive Transgenerational Plasticity. Proc. R. Soc. B Biol. Sci..

[43] Van Dooren T.J.M., Silveira A.B., Gilbault E., Jiménez-Gómez J.M., Martin A., Bach L., Tisné S., Quadrana L., Loudet O., Colot V. (2020). Mild Drought in the Vegetative Stage Induces Phenotypic, Gene Expression, and DNA Methylation Plasticity in *Arabidopsis* but No Transgenerational Effects. J. Exp. Bot..

[44] Liu N., Fromm M., Avramova Z. (2014). H3K27me3 and H3K4me3 Chromatin Environment at Super-Induced Dehydration Stress Memory Genes of *Arabidopsis thaliana*. Mol. Plant.

[45] Matzke M.A., Mosher R.A. (2014). RNA-Directed DNA Methylation: An Epigenetic Pathway of Increasing Complexity. Nat. Rev. Genet..

[46] Calarco J.P., Borges F., Donoghue M.T., Van Ex F., Jullien P.E., Lopes T., Gardner R., Berger F., Feijó J.A., Becker J.D. (2012). Reprogramming of DNA Methylation in Pollen Guides Epigenetic Inheritance via Small RNA. Cell.

[47] Borges F., Martienssen R.A. (2015). The Expanding World of Small RNAs in Plants. Nat. Rev. Mol. Cell Biol..

[48] Martinez G., Wolff P., Wang Z., Moreno-Romero J., Santos-González J., Conze L.L., DeFraia C., Slotkin R.K., Köhler C. (2018). Paternal easiRNAs Regulate Parental Genome Dosage in *Arabidopsis*. Nat. Genet..

[49] Muto A., Talarico E., D’Apice G., Di Marzo M., Moschin S., Nigris S., Babolin N., Greco E., Araniti F., Chiappetta A. (2024). Development of Pollinated and Unpollinated Ovules in *Ginkgo biloba*: Unravelling the Role of Pollen in Ovule Tissue Maturation. J. Exp. Bot..

[50] Heo J.B., Sung S. (2011). Vernalization-Mediated Epigenetic Silencing by a Long Intronic Noncoding RNA. Science.

[51] Chekanova J.A. (2015). Long Non-Coding RNAs and Their Functions in Plants. Curr. Opin. Plant Biol..

[52] Lucero L., Fonouni-Farde C., Crespi M., Ariel F. (2020). Long Noncoding RNAs Shape Transcription in Plants. Transcription.

[53] Greco M., Chiappetta A., Bruno L., Bitonti M.B. (2013). Effects of Light Deficiency on Genome Methylation in *Posidonia oceanica*. Mar. Ecol. Prog. Ser..

[54] Talarico E., Greco E., Chiappetta A., Araniti F., Bruno L. (2026). DNA Methylation Fine-Tunes Light-and Hormone-Responsive Growth Plasticity in *Arabidopsis* Seedlings. Int. J. Mol. Sci..

[55] Greco E., Talarico E., Guarasci F., Camoli M., Palermo A.M., Zambelli A., Chiappetta A., Araniti F., Bruno L. (2025). Epigenetic Mechanisms of Plant Adaptation to Cadmium and Heavy Metal Stress. Epigenomes.

[56] Talarico E., Greco E., Guarasci F., Araniti F., Chiappetta A., Bruno L. (2025). Epigenetic Regulation of Salt Stress Responses in Rice: Mechanisms and Prospects for Enhancing Tolerance. Epigenomes.

[57] Garro M., Greco E., Vannay G.J., Leonova A., Bruno L., Capella M. (2025). Non-CG DNA Methylation Represses *SDC* Expression to Modulate Hypocotyl Elongation during Thermormorphogenesis in Arabidopsis. J. Exp. Bot..

[58] Dar F.A., Mushtaq N.U., Saleem S., Rehman R.U., Dar T.U.H., Hakeem K.R. (2022). Role of Epigenetics in Modulating Phenotypic Plasticity against Abiotic Stresses in Plants. Int. J. Genom..

[59] Rudolf J., Tomovicova L., Panzarova K., Fajkus J., Hejatko J., Skalak J. (2024). Epigenetics and Plant Hormone Dynamics: A Functional and Methodological Perspective. J. Exp. Bot..

[60] Zhu Y. (2010). The Epigenetic Involvement in Plant Hormone Signaling. Chin. Sci. Bull..

[61] Yamamuro C., Zhu J.-K., Yang Z. (2016). Epigenetic Modifications and Plant Hormone Action. Mol. Plant.

[62] Fuentes-Merlos M.I., Bamba M., Sato S., Higashitani A. (2023). Self-Grafting-Induced Epigenetic Changes Leading to Drought Stress Tolerance in Tomato Plants. DNA Res..

[63] Yu Z., Zhang G., Teixeira da Silva J.A., Li M., Zhao C., He C., Si C., Zhang M., Duan J. (2021). Genome-Wide Identification and Analysis of DNA Methyltransferase and Demethylase Gene Families in *Dendrobium officinale* Reveal Their Potential Functions in Polysaccharide Accumulation. BMC Plant Biol..

[64] Sallam N., Moussa M. (2021). DNA Methylation Changes Stimulated by Drought Stress in ABA-Deficient Maize Mutant Vp10. Plant Physiol. Biochem..

[65] Santos A.S., Neves D.M., Santana-Vieira D.D.S., Almeida L.A.H., Costa M.G.C., Filho W.S.S., Pirovani C.P., Filho M.A.C., Ferreira C.F., Gesteira A.S. (2020). *Citrus* Scion and Rootstock Combinations Show Changes in DNA Methylation Profiles and ABA Insensitivity under Recurrent Drought Conditions. Sci. Hortic..

[66] Sallam N., Moussa M., Yacout M., Shakam H.M. (2022). Detection of DNA Methylation in DBF1 Gene of Maize Inbred W64A and Mutant *vp14* Exposed to Drought Stress. Cereal Res. Commun..

[67] Naderi S., Maali-Amiri R., Sadeghi L., Hamidi A. (2024). Physio-Biochemical and DNA Methylation Analysis of the Defense Response Network of Wheat to Drought Stress. Plant Physiol. Biochem..

[68] Gu D., Yang J., Wu S., Liao Y., Zeng L., Yang Z. (2021). Epigenetic Regulation of the Phytohormone Abscisic Acid Accumulation under Dehydration Stress during Postharvest Processing of Tea (*Camellia sinensis*). J. Agric. Food Chem..

[69] Tricker P.J., Rodríguez López C.M., Hadley P., Wagstaff C., Wilkinson M.J. (2013). Pre-Conditioning the Epigenetic Response to High Vapor Pressure Deficit Increases the Drought Tolerance of *Arabidopsis thaliana*. Plant Signal. Behav..

[70] Ackah M., Guo L., Li S., Jin X., Asakiya C., Aboagye E.T., Yuan F., Wu M., Essoh L.G., Adjibolosoo D. (2022). DNA Methylation Changes and Its Associated Genes in Mulberry (*Morus alba* L.) Yu-711 Response to Drought Stress Using MethylRAD Sequencing. Plants.

[71] Wang Q., Xu J., Pu X., Lv H., Liu Y., Ma H., Wu F., Wang Q., Feng X., Liu T. (2021). Maize DNA Methylation in Response to Drought Stress Is Involved in Target Gene Expression and Alternative Splicing. Int. J. Mol. Sci..

[72] Brunel-Muguet S., Vetukuri R.R., Testillano P.S. (2022). Epigenetics for Crop Adaptation to Climate Change. Physiol. Plant..

[73] Tirnaz S., Batley J. (2019). Epigenetics: Potentials and Challenges in Crop Breeding. Mol. Plant.

[74] Bhogale S., Mahajan A.S., Natarajan B., Rajabhoj M., Thulasiram H.V., Banerjee A.K. (2014). MicroRNA156: A Potential Graft-Transmissible microRNA That Modulates Plant Architecture and Tuberization in *Solanum tuberosum* ssp. *andigena*. Plant Physiol..

[75] Lafon-Placette C., Le Gac A.-L., Chauveau D., Segura V., Delaunay A., Lesage-Descauses M.-C., Hummel I., Cohen D., Jesson B., Le Thiec D. (2018). Changes in the Epigenome and Transcriptome of the Poplar Shoot Apical Meristem in Response to Water Availability Affect Preferentially Hormone Pathways. J. Exp. Bot..

[76] Surdonja K., Eggert K., Hajirezaei M.-R., Harshavardhan V.T., Seiler C., Von Wirén N., Sreenivasulu N., Kuhlmann M. (2017). Increase of DNA Methylation at the HvCKX2.1 Promoter by Terminal Drought Stress in Barley. Epigenomes.

[77] Sow M.D., Le Gac A.-L., Fichot R., Lanciano S., Delaunay A., Le Jan I., Lesage-Descauses M.-C., Citerne S., Caius J., Brunaud V. (2021). RNAi Suppression of DNA Methylation Affects the Drought Stress Response and Genome Integrity in Transgenic Poplar. New Phytol..

[78] Ahmad F., Farman K., Waseem M., Rana R.M., Nawaz M.A., Rehman H.M., Abbas T., Baloch F.S., Akrem A., Huang J. (2019). Genome-Wide Identification, Classification, Expression Profiling and DNA Methylation (5mC) Analysis of Stress-Responsive ZFP Transcription Factors in Rice (*Oryza sativa* L.). Gene.

[79] Lu X., Wang X., Chen X., Shu N., Wang J., Wang D., Wang S., Fan W., Guo L., Guo X. (2017). Single-Base Resolution Methylomes of Upland Cotton (*Gossypium hirsutum* L.) Reveal Epigenome Modifications in Response to Drought Stress. BMC Genom..

[80] Waseem M., Huang F., Wang Q., Aslam M.M., Abbas F., Ahmad F., Ashraf U., Hassan W., Fiaz S., Ye X. (2021). Identification, Methylation Profiling, and Expression Analysis of Stress-Responsive Cytochrome P450 Genes in Rice under Abiotic and Phytohormones Stresses. GM Crops Food.

[81] Pacenza M., Muto A., Chiappetta A., Mariotti L., Talarico E., Picciarelli P., Picardi E., Bruno L., Bitonti M.B. (2021). In *Arabidopsis thaliana* Cd Differentially Impacts on Hormone Genetic Pathways in the Methylation Defective Ddc Mutant Compared to Wild Type. Sci. Rep..

[82] Bruno L., Talarico E., Madeo M.L., Muto A., Minervino M., Araniti F., Bitonti M.B., Chiappetta A. (2021). Cadmium Affects Cell Niches Maintenance in *Arabidopsis thaliana* Post-Embryonic Shoot and Root Apical Meristem by Altering the Expression of WUS/WOX Homolog Genes and Cytokinin Accumulation. Plant Physiol. Biochem..

[83] Talarico E., Greco E., Araniti F., Chiappetta A., Bruno L. (2025). Non-CG DNA Methylation Regulates Root Stem Cell Niche Maintenance, Auxin Signaling, and ROS Homeostasis in *Arabidopsis* Under Cadmium Stress. Plants.

[84] Araniti F., Talarico E., Madeo M.L., Greco E., Minervino M., Álvarez-Rodríguez S., Muto A., Ferrari M., Chiappetta A., Bruno L. (2023). Short-Term Exposition to Acute Cadmium Toxicity Induces the Loss of Root Gravitropic Stimuli Perception through PIN2-Mediated Auxin Redistribution in *Arabidopsis thaliana* (L.) Heynh. Plant Sci..

[85] Bruno L., Araniti F., Talarico E., Greco E., Muto A., Pacenza M., Chiappetta A., Bitonti M.B. (2025). Transcriptomic and Metabolomic Analysis of the Main Stress-Related Pathways in the DNA Methylation-Defective *ddc* Mutant of *Arabidopsis thaliana* Exposed to Cadmium. Plant Biosyst.-Int. J. Deal. All Asp. Plant Biol..

[86] Bruno L., Talarico E., Cabeiras-Freijanes L., Madeo M.L., Muto A., Minervino M., Lucini L., Miras-Moreno B., Sofo A., Araniti F. (2021). Coumarin Interferes with Polar Auxin Transport Altering Microtubule Cortical Array Organization in *Arabidopsis thaliana* (L.) Heynh. Root Apical Meristem. Int. J. Mol. Sci..

[87] López-González D., Bruno L., Díaz-Tielas C., Lupini A., Aci M.M., Talarico E., Madeo M.L., Muto A., Sánchez-Moreiras A.M., Araniti F. (2023). Short-Term Effects of Trans-Cinnamic Acid on the Metabolism of *Zea mays* L. Roots. Plants.

[88] Seymen M., Burak Ö., Tanrıverdi, Yavuz D., Kıymacı G., Türkmen Ö., Kurtar E.S., Yavuz N., Kal Ü. (2024). Water Stress during Seedling Growth: Examining Physiological, Biochemical and Agronomic Dynamics in. *Cucumis Melo*. Pak. J. Agri. Sci..

[89] Ost C., Cao H.X., Nguyen T.L., Himmelbach A., Mascher M., Stein N., Humbeck K. (2023). Drought-Stress-Related Reprogramming of Gene Expression in Barley Involves Differential Histone Modifications at ABA-Related Genes. Int. J. Mol. Sci..

[90] Manh M.B., Ost C., Peiter E., Hause B., Krupinska K., Humbeck K. (2023). WHIRLY1 Acts Upstream of ABA-Related Reprogramming of Drought-Induced Gene Expression in Barley and Affects Stress-Related Histone Modifications. Int. J. Mol. Sci..

[91] Li J., Wei J., Song Y., Chen N., Ni B., Zhang J., He C. (2023). Histone H3K9 Acetylation Modulates Gene Expression of Key Enzymes in the Flavonoid and Abscisic Acid Pathways and Enhances Drought Resistance of Sea Buckthorn. Physiol. Plant..

[92] Ma X., Zhang B., Liu C., Tong B., Guan T., Xia D. (2017). Expression of a Populus Histone Deacetylase Gene 84KHDA903 in Tobacco Enhances Drought Tolerance. Plant Sci..

[93] Han Y., Haouel A., Georgii E., Priego-Cubero S., Wurm C.J., Hemmler D., Schmitt-Kopplin P., Becker C., Durner J., Lindermayr C. (2023). Histone Deacetylases HD2A and HD2B Undergo Feedback Regulation by ABA and Modulate Drought Tolerance via Mediating ABA-Induced Transcriptional Repression. Genes.

[94] Wang X., Pan C., Long J., Bai S., Yao M., Chen J., Sun G., Fan Y., Wang Z., Liu F. (2022). Genome-Wide Identification of the Jumonji C Domain- Containing Histone Demethylase Gene Family in Wheat and Their Expression Analysis under Drought Stress. Front. Plant Sci..

[95] Yu X., Gao Q., Chen G., Guo J.-E., Guo X., Tang B., Hu Z. (2018). SlHDA5, a Tomato Histone Deacetylase Gene, Is Involved in Responding to Salt, Drought, and ABA. Plant Mol. Biol. Rep..

[96] Guo J.-E., Wang H., Yang Y., Li J., Zhu Z. (2023). Histone Deacetylase Gene SlHDA3 Is Involved in Drought and Salt Response in Tomato. Plant Growth Regul..

[97] Xin X., Su T., Li P., Wang W., Zhao X., Yu Y., Zhang D., Yu S., Zhang F. (2021). A Histone H4 Gene Prevents Drought-Induced Bolting in Chinese Cabbage by Attenuating the Expression of Flowering Genes. J. Exp. Bot..

[98] Gao W., Li M., Yang S., Gao C., Su Y., Zeng X., Jiao Z., Xu W., Zhang M., Xia K. (2022). MiR2105 and the Kinase OsSAPK10 Co-Regulate OsbZIP86 to Mediate Drought-Induced ABA Biosynthesis in Rice. Plant Physiol..

[99] Candar-Cakir B., Arican E., Zhang B. (2016). Small RNA and Degradome Deep Sequencing Reveals Drought-and Tissue-Specific Micrornas and Their Important Roles in Drought-Sensitive and Drought-Tolerant Tomato Genotypes. Plant Biotechnol. J..

[100] Wang Y., Dai J., Chen R., Song C., Wei P., Cai Y., Wang Y., Han B. (2022). MiRNA-Based Drought Regulation in the Important Medicinal Plant *Dendrobium huoshanense*. J. Plant Growth Regul..

[101] Chen J., Li L. (2018). Multiple Regression Analysis Reveals MicroRNA Regulatory Networks in *Oryza sativa* under Drought Stress. Int. J. Genom..

[102] Esmaeili F., Shiran B., Fallahi H., Mirakhorli N., Budak H., Martínez-Gómez P. (2017). In Silico Search and Biological Validation of microRNAs Related to Drought Response in Peach and Almond. Funct. Integr. Genom..

[103] Saminathan T., Alvarado A., Lopez C., Shinde S., Gajanayake B., Abburi V.L., Vajja V.G., Jagadeeswaran G., Reddy U.K., Nimmakayala P. (2019). Elevated Carbon Dioxide and Drought Modulate Physiology and Storage-Root Development in Sweet Potato by Regulating microRNAs. Funct. Integr. Genom..

[104] Ahmad H.M., Wang X., Ijaz M., Mahmood-Ur-Rahman, Oranab S., Ali M.A., Fiaz S. (2022). Molecular Aspects of microRNAs and Phytohormonal Signaling in Response to Drought Stress: A Review. Curr. Issues Mol. Biol..

[105] Zhang F., Yang J., Zhang N., Wu J., Si H. (2022). Roles of microRNAs in Abiotic Stress Response and Characteristics Regulation of Plant. Front. Plant Sci..

[106] Anwar A., Zhang S., He L., Gao J. (2022). Understanding the Physiological and Molecular Mechanism of Salinity Stress Tolerance in Plants. Not. Bot. Horti Agrobot. Cluj.-Napoca.

[107] Kladde M.P., Simpson R.T. (1994). Positioned Nucleosomes Inhibit Dam Methylation in vivo. Proc. Natl. Acad. Sci. USA.

[108] Kościelniak P., Glazińska P., Kȩsy J., Zadworny M. (2021). Formation and Development of Taproots in Deciduous Tree Species. Front. Plant Sci..

[109] Fard E.M., Bakhshi B., Farsi M., Kakhki A.M., Nikpay N., Ebrahimi M.A., Mardi M., Salekdeh G.H. (2017). MicroRNAs Regulate the Main Events in Rice Drought Stress Response by Manipulating the Water Supply to Shoots. Mol. Biosyst..

[110] Jiao P., Ma R., Wang C., Chen N., Liu S., Qu J., Guan S., Ma Y. (2022). Integration of mRNA and microRNA Analysis Reveals the Molecular Mechanisms Underlying Drought Stress Tolerance in Maize (*Zea mays* L.). Front. Plant Sci..

[111] He Z., Liu C., Zhang Z., Wang R., Chen Y. (2022). Integration of mRNA and miRNA Analysis Reveals the Differentially Regulatory Network in Two Different *Camellia oleifera* Cultivars under Drought Stress. Front. Plant Sci..

[112] Deng M., Cao Y., Zhao Z., Yang L., Zhang Y., Dong Y., Fan G. (2017). Discovery of MicroRNAs and Their Target Genes Related to Drought in Paulownia “Yuza 1” by High-Throughput Sequencing. Int. J. Genom..

[113] Yang X., Liu C., Niu X., Wang L., Li L., Yuan Q., Pei X. (2022). Research on lncRNA Related to Drought Resistance of Shanlan Upland Rice. BMC Genom..

[114] Carbone F., Bruno L., Perrotta G., Bitonti M.B., Muzzalupo I., Chiappetta A. (2019). Identification of miRNAs Involved in Fruit Ripening by Deep Sequencing of *Olea europaea* L. Transcriptome. PLoS ONE.

[115] Chakraborty A., Viswanath A., Malipatil R., Rathore A., Thirunavukkarasu N. (2020). Structural and Functional Characteristics of miRNAs in Five Strategic Millet Species and Their Utility in Drought Tolerance. Front. Genet..

[116] Chen R., Li M., Zhang H., Duan L., Sun X., Jiang Q., Zhang H., Hu Z. (2019). Continuous Salt Stress-Induced Long Non-Coding RNAs and DNA Methylation Patterns in Soybean Roots. BMC Genom..

[117] Tombuloglu G. (2022). Drought-Responsive miRNAs in Plants: A Review. Int. J. Innov. Eng. Appl..

[118] Kaur S., Seem K., Kumar D., Kumar S., Kaundal R., Mohapatra T. (2024). Biogenesis to Functional Significance of microRNAs under Drought Stress in Rice: Recent Advances and Future Perspectives. Plant Stress.

[119] Cai Y., Cheng L., Liu X., Li R., Liu Y., Ge S., Wang S., Liu J., Tan C., Meng S. (2025). SlALKBH9B Is Involved in Drought-Induced Flower Drop by Regulating Ethylene Production. Hortic. Res..

[120] Hou N., Li C., He J., Liu Y., Yu S., Malnoy M., Tahir M.M., Xu L., Ma F., Guan Q. (2022). MdMTA-Mediated m6A Modification Enhances Drought Tolerance by Promoting mRNA Stability and Translation Efficiency of Genes Involved in Lignin Deposition and Oxidative Stress. New Phytol..

[121] Zhu C., Zhang S., Zhou C., Xie S., Chen G., Tian C., Xu K., Lin Y., Lai Z., Guo Y. (2021). Genome-Wide Investigation of N6-Methyladenosine Regulatory Genes and Their Roles in Tea (*Camellia sinensis*) Leaves During Withering Process. Front. Plant Sci..

[122] Li B., Zhang M., Sun W., Yue D., Ma Y., Zhang B., Duan L., Wang M., Lindsey K., Nie X. (2023). N6-Methyladenosine RNA Modification Regulates Cotton Drought Response in a Ca^2+^ and ABA-Dependent Manner. Plant Biotechnol. J..

[123] Ganguly D.R., Li Y., Bhat S.S., Tiwari S., Ng P.J., Gregory B.D., Sunkar R. (2025). MRNA ADENOSINE METHYLASE Promotes Drought Tolerance through N^6^-Methyladenosine-Dependent and Independent Impacts on mRNA Regulation in *Arabidopsis*. New Phytol..

[124] Guarino F., Cicatelli A., Castiglione S., Agius D.R., Orhun G.E., Fragkostefanakis S., Leclercq J., Dobránszki J., Kaiserli E., Lieberman-Lazarovich M. (2022). An Epigenetic Alphabet of Crop Adaptation to Climate Change. Front. Genet..

[125] Yu L., Alariqi M., Li B., Hussain A., Zhou H., Wang Q., Wang F., Wang G., Zhu X., Hui F. (2024). CRISPR/dCas13(Rx) Derived RNA N^6^-Methyladenosine (m^6^A) Dynamic Modification in Plant. Adv. Sci..

[126] Hou Q., Wan X. (2021). Epigenome and Epitranscriptome: Potential Resources for Crop Improvement. Int. J. Mol. Sci..

[127] Han R., Nguyen T.K.H., Yang Z., Kang H. (2025). ECT10 Is an m^6^A Reader That Negatively Regulates the Drought Stress Response in *Arabidopsis* by Modulating mRNA Stability. Plant J..

[128] Adhikari D. (2023). Role of Epigenetics and the High-Throughput Sensing Techniques to Detect Stress Adaptation Mechanisms in Crop Plants: A Mini Review. Innov. Agric..

[129] Minnoye L., Marinov G.K., Krausgruber T., Pan L., Marand A.P., Secchia S., Greenleaf W.J., Furlong E.E.M., Zhao K., Schmitz R.J. (2021). Chromatin Accessibility Profiling Methods. Nat. Rev. Methods Primers.

[130] Li S., Tollefsbol T.O. (2021). DNA Methylation Methods: Global DNA Methylation and Methylomic Analyses. Methods.

[131] Shaik R., Ramakrishna W. (2012). Bioinformatic Analysis of Epigenetic and microRNA Mediated Regulation of Drought Responsive Genes in Rice. PLoS ONE.

[132] Garg R., Narayana Chevala V.V.S., Shankar R., Jain M. (2015). Divergent DNA Methylation Patterns Associated with Gene Expression in Rice Cultivars with Contrasting Drought and Salinity Stress Response. Sci. Rep..

[133] Zheng X., Chen L., Xia H., Wei H., Lou Q., Li M., Li T., Luo L. (2017). Transgenerational Epimutations Induced by Multi-Generation Drought Imposition Mediate Rice Plant’s Adaptation to Drought Condition. Sci. Rep..

[134] Mundade R., Ozer H.G., Wei H., Prabhu L., Lu T. (2014). Role of ChIP-Seq in the Discovery of Transcription Factor Binding Sites, Differential Gene Regulation Mechanism, Epigenetic Marks and Beyond. Cell Cycle.

[135] Song J., Henry H., Tian L. (2020). Drought-Inducible Changes in the Histone Modification H3K9ac Are Associated with Drought-Responsive Gene Expression in *Brachypodium distachyon*. Plant Biol..

[136] Dasgupta P., Prasad P., Bag S.K., Chaudhuri S. (2022). Dynamicity of Histone H3K27ac and H3K27me3 Modifications Regulate the Cold-Responsive Gene Expression in *Oryza sativa* L. ssp. *indica*. Genomics.

[137] Zong W., Yang J., Fu J., Xiong L. (2020). Synergistic Regulation of Drought-Responsive Genes by Transcription Factor OsbZIP23 and Histone Modification in Rice. J. Integr. Plant Biol..

[138] Zhao W., Wang X., Zhang Q., Zheng Q., Yao H., Gu X., Liu D., Tian X., Wang X., Li Y. (2022). H3K36 Demethylase JMJ710 Negatively Regulates Drought Tolerance by Suppressing MYB48-1 Expression in Rice. Plant Physiol..

[139] Wang S., He J., Deng M., Wang C., Wang R., Yan J., Luo M., Ma F., Guan Q., Xu J. (2022). Integrating ATAC-Seq and RNA-Seq Reveals the Dynamics of Chromatin Accessibility and Gene Expression in Apple Response to Drought. Int. J. Mol. Sci..

[140] Mladenov P., Wang X., Yang Z., Djilianov D., Deng X. (2023). Dynamics of Chromatin Accessibility and Genome Wide Control of Desiccation Tolerance in the Resurrection Plant *Haberlea rhodopensis*. BMC Plant Biol..

[141] Yang J., Chang Y., Qin Y., Chen D., Zhu T., Peng K., Wang H., Tang N., Li X., Wang Y. (2020). A Lamin-like Protein OsNMCP1 Regulates Drought Resistance and Root Growth through Chromatin Accessibility Modulation by Interacting with a Chromatin Remodeller OsSWI3C in Rice. New Phytol..

[142] Chenarani N., Emamjomeh A., Allahverdi A., Mirmostafa S., Afsharinia M.H., Zahiri J. (2021). Bioinformatic Tools for DNA Methylation and Histone Modification: A Survey. Genomics.

[143] Yang X., Sohail H., Noor I., Costa F.C.L., Zhong S., Zhang L., Chen X. (2025). Epigenetic Crop Improvement: Integrating ENCODE Strategies into Horticultural Breeding. Hortic. Res..

[144] Sheoran S., Kaur Y., Kumar S., Shukla S., Rakshit S., Kumar R. (2022). Recent Advances for Drought Stress Tolerance in Maize (*Zea mays* L.): Present Status and Future Prospects. Front. Plant Sci..

[145] Ghaffar W., Qaseem Q.E.A., Mustafa G., Khan M.S., Riaz R. (2025). Role of AI and Big Data for Managing Plant Stress in Smart Agriculture. Integr. Plant Biotechnol..

[146] Mushtaq M., Ahmad Dar A., Skalicky M., Tyagi A., Bhagat N., Basu U., Bhat B.A., Zaid A., Ali S., Dar T.-U.-H. (2021). CRISPR-Based Genome Editing Tools: Insights into Technological Breakthroughs and Future Challenges. Genes.

[147] Corem S., Doron-Faigenboim A., Jouffroy O., Maumus F., Arazi T., Bouché N. (2018). Redistribution of CHH Methylation and Small Interfering RNAs across the Genome of Tomato Ddm1 Mutants. Plant Cell.

[148] Wang X., Guo Y., Wang Y., Peng Y., Zhang H., Zheng J. (2024). ZmHDT103 Negatively Regulates Drought Stress Tolerance in Maize Seedlings. Agronomy.

[149] Thakore P.I., D’Ippolito A.M., Song L., Safi A., Shivakumar N.K., Kabadi A.M., Reddy T.E., Crawford G.E., Gersbach C.A. (2015). Highly Specific Epigenome Editing by CRISPR-Cas9 Repressors for Silencing of Distal Regulatory Elements. Nat. Methods.

[150] Kang J.G., Park J.S., Ko J.-H., Kim Y.-S. (2019). Regulation of Gene Expression by Altered Promoter Methylation Using a CRISPR/Cas9-Mediated Epigenetic Editing System. Sci. Rep..

[151] Lee J.H., Mazarei M., Pfotenhauer A.C., Dorrough A.B., Poindexter M.R., Hewezi T., Lenaghan S.C., Graham D.E., Stewart C.N. (2020). Epigenetic Footprints of CRISPR/Cas9-Mediated Genome Editing in Plants. Front. Plant Sci..

[152] Devesa-Guerra I., Morales-Ruiz T., Pérez-Roldán J., Parrilla-Doblas J.T., Dorado-León M., García-Ortiz M.V., Ariza R.R., Roldán-Arjona T. (2020). DNA Methylation Editing by CRISPR-Guided Excision of 5-Methylcytosine. J. Mol. Biol..

[153] Vasquez J.-J., Wedel C., Cosentino R.O., Siegel T.N. (2018). Exploiting CRISPR–Cas9 Technology to Investigate Individual Histone Modifications. Nucleic Acids Res..

[154] Crampton M., Sripathi V.R., Hossain K., Kalavacharla V. (2016). Analyses of Methylomes Derived from Meso-American Common Bean (*Phaseolus vulgaris* L.) Using MeDIP-Seq and Whole Genome Sodium Bisulfite-Sequencing. Front. Plant Sci..

[155] Hua Y., Zhang C., Shi W., Chen H. (2019). High-Throughput Sequencing Reveals microRNAs and Their Targets in Response to Drought Stress in Wheat (*Triticum aestivum* L.). Biotechnol. Biotechnol. Equip..

[156] Amara U., Shoaib Y., Kang H. (2022). ALKBH9C, a Potential RNA m6A Demethylase, Regulates the Response of *Arabidopsis* to Abiotic Stresses and Abscisic Acid. Plant Cell Environ..

[157] Lin H., Shi T., Zhang Y., He C., Zhang Q., Mo Z., Pan W., Nie X. (2023). Genome-Wide Identification, Expression and Evolution Analysis of m6A Writers, Readers and Erasers in *Aegilops_tauschii*. Plants.

[158] Bruno L., Spadafora N.D., Iaria D., Chiappetta A., Bitonti M.B. (2014). Developmental Stimuli and Stress Factors Affect Expression of *ClGLP1*, an Emerging Allergen-Related Gene in *Citrus limon*. Plant Physiol. Biochem..

